# Potential in vitro anti-allergic, anti-inflammatory and cytotoxic activities of ethanolic extract of *Baliospermum montanum* root, its major components and a validated HPLC method

**DOI:** 10.1186/s12906-019-2449-0

**Published:** 2019-02-12

**Authors:** Weerachai Pipatrattanaseree, Arunporn Itharat, Nichamon Mukkasombut, Ubonwan Saesiw

**Affiliations:** 1 Department of Applied Thai Traditional Medicine, Faculty of Medicine, Thammasat University, Klongluang, Pathumthani 12120 Thailand; 2 Center of Excellence on Applied Thai Traditional Medicine Researches, Faculty of Medicine, Thammasat University, Klongluang, Pathumthani 12120 Thailand

**Keywords:** Anti-allergy, Anti-inflammation, Cytotoxicity, *Baliospermum montanum*, Propiophenones

## Abstract

**Background:**

The root of *Baliospermum montanum* has been used as an ingredient of traditional Thai medicines for the treatments of several diseases including itching eczema, muscle and joint inflammation, and cancer. Few studies have been done on phytochemical components of this root. In this study, we isolated major compounds of the crude ethanolic extract of *B. montanum* root and developed and validated a high performance liquid chromatographic (HPLC) method for the determination of its major components. We then investigated anti-allergic, anti-inflammatory and cytotoxic activities of the extract.

**Methods:**

The aims of this study were to investigate in vitro activities including inhibitory effect of β-hexosaminidase released from RBL-2H3 cells, inhibition of nitric oxide (NO) production from RAW 264.7 cells and cytotoxic activity against cancerous liver cell lines (HepG2 and KKU M156) by using sulforhodamine B (SRB) assay. Isolation of major components was conducted by using column chromatographic method. Isolated major compounds were analyzed by using high performance liquid chromatography (HPLC).

**Results:**

The crude extract exhibited the highest cytotoxic activity, with IC_50_ less than 1 μg/mL, while its anti-allergy and anti-inflammation were also potent with IC_50_ less than 6 μg/mL. Three propiophenones isolated from *B. montanum* root exhibited moderate cytotoxic activities (IC_50_ > 20 μg/mL). Two of the propiophenones found were major components that can be detected by HPLC. The developed and validated HPLC method showed good accuracy, precision, and linearity.

**Conclusion:**

The results of this study suggested that ethanolic extract of of *B.montanum* root can be a potential source of anti-allergy, anti-inflammation, and anti-cancer compounds. The isolated compounds can serve as markers when *B. montanum* is used in herbal remedies but not as overall responsive markers. The HPLC method developed may be useful for quality control in the production of the extract and for further formulation developments. However, investigation of several associated biological activities is necessary before the development can proceed further. Minor active compounds should be isolated and a more sensitive analytical method should be developed to detail the key responsive components of the ethanolic extract of *B. montanum* root.

**Electronic supplementary material:**

The online version of this article (10.1186/s12906-019-2449-0) contains supplementary material, which is available to authorized users.

## Background

*Baliospermum montanum* (Willd.) Muell-Arg is a leafy stout monoecious undershrub plant, a member of Euphorbiaceae. It grows naturally in middle- and south-east Asia and has been used as a traditional medicine in several countries. In Ayurvedic medicine, *B. montanum* root is used to treat skin diseases, worm infection, gastrointestinal tract diseases, and itching [1]. In traditional Thai medicines (TTM), *B. montanum* root is an important ingredient in several remedies such as ‘Sahastara’ for muscle and joint pain [2] and ‘Benja-Amarit’ for liver cancer [3]. It is also used for the treatment of eczema itches [4].

The biological activities of *B. montanum* root have been investigated. Kakatum and co-workers reported in vitro anti-inflammatory effect of *B. montanum* root, a component in a Thai polyherbal preparation called “Sahastara” used for the treatment of muscle and joint pain [5]. Ogura and co-workers reported in vitro cytotoxicity of the isolated compounds from the root against leukemia P-388 cells [6]. In an in vivo study, Venkatesh and co-worker reported that leaves of *B. montanum* inhibited the degranulation of mast cells in systemic anaphylaxis model [7]. Other researchers reported anti-oxidant [5], antimicrobial activities [8], immunomodulatory activities [9] and hepatoprotective properties of *B. montanum* [10, 11]. Phytochemical screening revealed that chemical constituents in the root of *B. montanum* were steroids, terpenoids, glycosides, saponins, alkaloids, flavonoids, phenolic compounds, tannins, sugars, and several other minor components [6–8].

Based on the traditional uses and scientific evidences above, we selected in vitro biological effects as preliminary models to investigate the associated biological properties of the ethanolic extract of *B. montanum* root. Exhaustive search reveals no reports on inhibitory effect of β-hexosaninidase released from RBL-2H3 cells and cytotoxic activity against cancerous liver cells of *B. montanum* crude extract. Neither were reports on its major components and their analytical methods found. This present study aimed at investigating anti-allergic activity by determining the inhibitory effect on β-hexosaminidase released from RBL-2H3 cells, anti-inflammatory activity by detecting the inhibition of nitric oxide (NO) production from RAW 264.7 cells and cytotoxic activity against cancerous liver cell lines by using sulforhodamine B (SRB) assay. The isolation of major components and evaluation of their activities were also conducted. Subsequently, a quantitative HPLC method for the determination of its major components was developed and validated.

## Methods

### Plant materials and preparations

*B.montanum* roots were collected from Lampoon province, Thailand, in January 2016 and authenticated by Department of Applied Thai traditional Medicines, Thammasat University. The vouchered specimen (SKP-071021301) was deposited at the herbarium of Southern Center of Thai Medicinal Plants, Faculty of Pharmaceutical Sciences, Prince of Songkla University, Songkhla, Thailand.

Roots of *B. montanum* were washed, sliced thinly and dried in a hot air oven at the temperature not more than 45 °C, and ground to coarse powder. One kg of the dried root was macerated with 95% ethanol (3 × 3 days). The extracts were filtered, combined and concentrated with a rotary evaporator under a reduced pressure at 45 °C.

### Reagents and chemicals

Analytical grade organic solvents: hexane, chloroform, ethyl acetate, methanol, acetonitrile and methanol (HPLC grade) were purchased from RCI LabScan (Bangkok, Thailand). Purified water was prepared by Milli Q® system from Millipore (Bedford, MA, USA).

Chlorpheniramine, prednisolone, and vincristine were purchased from Sigma (MO, USA). Chemical reference substances used for method validation, compounds 1 and 2, were isolated from the crude ethanolic extract and identified by the spectroscopic method including UV, MS, and NMR. The purities of isolated compounds were determined to be more than 98% by using HPLC analysis.

Rat basophilic leukemia cell line (RBL-2H3: ATCC CRL-2256™), murine macrophage leukemia cell line (RAW 264.7: ATCC® TIB-71™) and hepatocellular carcinoma cell line (HepG2: ATCC® HB-8065™) were purchased from American Type Culture Collection (ATCC®, VA, USA). Cholangiocarcinoma cell line (KKU-M156) was obtained from Dr. Piti Aungareewithaya and Prof. Dr. Veeraphol Kukongviriyapan, Faculty of Medicine, Khon-Kaen University. Normal human keratinocyte immortal cell line (HaCaT) was purchased from CLS cell line service (No. 300493-SF).

Fetal bovine serum (FBS) and trypsin-EDTA were purchased from Gibco® (OK, USA). Minimum essential medium (MEM), Dulbecco's Modified Eagle's Medium (DMEM), Roswell Park Memorial Institute medium 1640 (RPMI-1640), penicillin-streptomycin (P/S), and phosphate buffer saline (PBS) were purchased from Biochrom (MA, Germany). Nutrient Mixture F-12 Ham medium (HAM F-12), 3-(4, 5-dimethyl-2-thiazolyl)-2, 5-diphenyl-2*H*-tetrazolium bromide (MTT), Sulforhodamine B (SRB), 4-Nitrophenyl N-acetyl-β-D-glucosaminide (PNAG), anti-dinitrophenylated bovine albumin (DNP-BSA) and anti-dinitrophenyl immunoglobulin E (Anti-DNP IgE) were purchased from Sigma (MO, USA). Dimethyl sulfoxide (DMSO) was purchased from Fluka (Munich, Germany).

### Instruments

HPLC analysis was performed on an Agilent® 1200 HPLC system (Agilent Technologies, USA) composing of a solvent degasser (G1322A), a quaternary solvent pump (G1311A), an autosampler (G1329A), a column oven (G1316A) and a photodiode array detector (G1315D). The chromatographic data were processed by the Chemstation® software revision B.04.01 SP1. The ESI-HRMS were performed either on Shimadzu ion trap-time of flight spectrometer (IT-TOF) (Shimadzu Corporation, Japan) or Bruker microTOF (Bruker company, USA). Nuclear magnetic resonance (NMR) spectra were recorded either on Bruker Avance DRX-500 NMR spectrometer (Bruker Company, USA) or Varian Unity Inova 500 MHz (Variance, USA) using tetramethylsilane as internal standard.

### Anti-allergic activity

Inhibitory activity on *β*-hexosaminidase released from rat basophilic leukemia cells (RBL-2H3) was evaluated following the report of Juckmetha and co-workers [12]. RBL-2H3 cells were cultured in MEM supplemented with 15% FBS, penicillin/streptomycin (each 100 μg/mL). Cells were seeded in 24-well plate with the concentration of 5 × 10^5^ cells/well and incubated in 5% CO_2_ at 37 °C for 1.5 h. RBL-2H3 were sensitized with 0.45 μg/mL of Anti-DNP IgE and incubated at 37 °C in 5% CO_2_ for 24 h. Cells were then washed with 400 μL of Siraganian buffer (Buffer A) [119 mM NaCl, 5 mM KCl, 5.6 mM glucose, 0.4 mM MgCl_2_, 1 mM CaCl_2_, 25 mM piperazine-*N,N′*-bis (2-ethanesulfonic acid) (PIPES), 0.1% BSA, and 40 mM NaOH, pH 7.2]. Subsequently, 160 μL of buffer A was added into the wells and incubated at 37 °C for 10 min. The stock solution of the sample was diluted in DMSO and the working samples were diluted with buffer A. The concentrations of the working samples were 0.1 μg/mL, 1 μg/mL, 10 μg/mL and 50 μg/mL. The serially diluted samples (20 μL) were added to each well and incubated for 10 min. The reaction of antigen-antibody was obtained by adding 20 μL of antigen (DNP-BSA) to produce a final concentration of 10 μg/mL and incubated at 37 °C for 20 min. The amount of *β-*hexosaminidase released from the degranulation was determined by adding 50 μL of the substrate, PNAG (1 mM in 0.1 M citrate buffer, pH 4.5). The enzyme reaction was stopped by adding 200 μL of 0.1 M carbonate buffer pH 10.0. The product of enzyme reaction, *p*-nitrophenol, was analyzed by measuring absorbance at wavelength 405 nm. Chlorpheniramine was used as positive control. The percentage of inhibition was calculated by the following equation.

$$ \mathrm{Inhibition}\ \left(\%\right)=\left[1-\frac{\left(\mathrm{T}-\mathrm{B}-\mathrm{N}\right)}{\left(\mathrm{C}-\mathrm{B}-\mathrm{N}\right)}\right]\times 100 $$

Where

- C = Optical density of control: [+] DNP-BSA, [−] Test sample;
- T = Optical density of test sample: [+] DNP-BSA, [+] Test sample;
- B = Optical density of blank: [+] DNP-BSA, [+] Test sample;
- N = Optical density of normal: [−] DNP-BSA, [−] Test sample.

### Anti-inflammatory activity

The inhibitory activity of crude extracts and isolated compounds on NO production of LPS-induced RAW 264.7 cells was investigated according to a previously reported method [13]. RAW 264.7 cells were cultured in RPMI medium supplemented with 10% FBS, penicillin/streptomycin (each 100 units/mL) and incubated at 37 °C in 5% CO_2_ with 95% humidity. The cells were seeded into 96-well plate (1 × 10^5^ cells per well) and incubated for 24 h. Subsequently, the cells were induced by LPS at the final concentration of 2 ng/mL and the serially diluted samples (0.1 μg/mL, 1 μg/mL, 10 μg/mL and 50 μg/mL) were added. The plate was incubated for 24 h before being tested for NO accumulation and cell viability. Prednisolone was used as positive control. The accumulated NO released from the LPS*-*induced RAW 264.7 cells was determined by using Griess’s reagent (1% sulfanilamide and 0.1% N-(1- naphthyl) ethylenediamine dihydrochloride in 2.5% phosphoric acid). The supernatant in each well (100 μL) was transferred to another 96-well plate and 100 μL of Griess’s reagent was added. The optical density of reaction was measured at 570 nm. The inhibitory activity (%) of NO production was calculated by the following equation and the IC_50_ values were calculated.

$$ \mathrm{Inhibition}\kern0.24em \left(\%\right)=\left[\frac{{\mathrm{OD}}_{\mathrm{control}}\hbox{-} {\mathrm{OD}}_{\mathrm{sample}}}{{\mathrm{OD}}_{\mathrm{control}}}\right]\times 100 $$

The viability of the LPS treated RAW 264.7 cells were colorimetrically determined by using 3-(4, 5-dimethyl-2-thiazolyl)-2, 5-diphenyl-2*H*-tetrazolium bromide (MTT) method. MTT solution (10 μL) was added into each well and the plate was incubated for 2 h. The remained formazan crystal in each well, after removing the medium, was dissolved by 0.04 M HCl in isopropanol (100 μL). The optical density was measured by using microplate reader at wavelength 570 nm. The cell viability of more than 70% (Table 2) indicated that the inhibitory effect was not derived from cell death.

### Cytotoxic activity

The crude extracts and isolated compounds were evaluated for cytotoxic activity by using sulforhodamine B (SRB) assay against two cancerous liver cells (HepG2 and KKU-M156) and a normal cells (HaCaT). The SRB protocol has been described previously [14]. HepG2 was cultured in MEM; KKU-M156 was cultured in HAM F-12 media and HaCaT was cultured in DMEM. All cultured media were supplemented with 10% FBS, penicillin/streptomycin (each 100 units/mL). All cells were incubated in 5% CO_2_ at 37 °C with 95% humidity. Based on the growth curve, each cell was seeded into 96-well plate (100 μL per well) with optimal density (HepG2: 3 × 10^3^ cells per well, KKU-M156: 2 × 10^3^ cells per well and HaCaT: 8 × 10^3^ cells per well). Seeded cells were incubated at 37 °C in a 5% CO_2_ with 95% humidity for 24 h. Cells were then treated with 100 μL of different concentrations of sample solution (0.1 μg/mL, 1 μg/mL, 10 μg/mL and 50 μg/mL). DMSO at the same concentration as the sample (2% *v*/v) was used as the control solvent. Cell cultures were incubated for the exposure time of 72 h. After the exposure time, the medium was removed and the plate was washed with 200 μL phosphate buffer saline (PBS) and 200 μL fresh medium was added into each well. The plates were then incubated for 72 h for the recovery period. The cells were then fixed by adding 100 μL of 40% trichloroacetic acid and the plates left at 4 °C for 45 min. After the plates were washed, 50 μL of SRB solution (0.4% *w*/*v* in acetic acid) was added into each well and the cells were allowed to be strained for 30 min. The stained cells were dissolved by using 100 μL of 10 mM Tris base pH 10.5. The %inhibition was calculated using the following equation.

$$ \mathrm{Inhibition}\;\left(\%\right)=\left(\frac{{\mathrm{OD}}_{\mathrm{control}}\hbox{-} {\mathrm{OD}}_{\mathrm{sample}}}{{\mathrm{OD}}_{\mathrm{control}}}\right)\times 100 $$

### Isolation of major constituents from the root of *B. montanum*

Pure compounds were isolated by using silica column chromatography (CC) and HPLC techniques. Ethanolic extract of *B. montanum* root (40 g) was subjected to a silica gel column (49–63 μm, 200 g). The extract was eluted sequentially by using 100% hexane (700 mL), hexane/ chloroform 7:3 (700 mL), 3:7 (700 mL), 100% chloroform (700 mL), chloroform/ethyl acetate 7:3 (700 mL), 3:7 (700 mL), 100% ethyl acetate (700 mL), ethyl acetate/ methanol 7:3 (700 mL), 3:7 (700 mL) and 100% methanol (700 mL). Ten obtained fractions, namely FF01 to FF10, were analyzed by using the HPLC method described below.

Fractions FF03 and FF04 were combined (6.8 g) and subjected to second silica gel column (90 g). Polarity gradient elutants were hexane, chloroform, ethyl acetate, and methanol, consecutively. Fractions were collected according to the results from TLC monitoring. Eight fractions obtained from the second CC were named SF01 to SF08.

Fraction SF03 (536 mg) was subjected to third silica gel column (24 g) and eluted by polarity gradient mobile phases from hexane to chloroform and ending with ethyl acetate. Fractions were collected according to the results from TLC monitoring. Eight fractions obtained from the third CC were named TF01 to TF08.

Fraction TF02 (10.8 mg) was further fractionated by semi-preparative HPLC (Zorbax® 5 μm, 10 mm × 250 mm, 5 μm; 40% H_2_O in methanol, flow rate 3.0 ml/min) to yield compound 1 (4.0 mg) and compound 3 (1.3 mg). Fraction TF03 (40.9 mg) was subjected to semi-preparative HPLC (Zorbax® 5 μm, 250 mm × 10 mm; 50% H_2_O in acetonitrile, flow rate 3.0 ml/min) to yield compound 1 (9.8 mg), and compound 2 (30.1 mg). All isolated compounds were characterized by using ^1^H NMR, ^13^C NMR, 2D NMR, and MS.

### High performance liquid chromatography analysis

#### Preparation of samples and standard solutions

Sample solutions of ethanolic extracts of *B. montanum* root and their CC fractions were prepared at the concentrations of 10 mg/mL and 1 mg/mL, respectively. Samples were weighed accurately and dissolved with a mixture of methanol:chloroform (9.5:0.5) with the aid of sonication. Solutions of the isolated compounds were prepared at the concentration of 1 mg/mL in methanol for the determination of their purity. Different concentrations of the isolated standard solutions were prepared in methanol according to the linear range. All prepared solutions were filtered through 0.45 μm membrane before injecting into the HPLC system.

#### Chromatographic system

Major components of the crude extract were separated along the C18 reverse phase column (Zorbax® C18, 4.6 × 150, 5 μm). Gradient mobile phase composing of water (A) and acetonitrile (B) was programmed as follows: 0–10 min, 20%B; 10–15 min, 200%B - 65%B; 15.1–20 min, 20%B. The flow rate was set at 1.0 mL/min. Samples were injected into the HPLC system and detected with diode array detector using the wavelength of 280 nm.

### Method validation

The developed quantitative method for the determination of compounds 1 and 2 in ethanolic extract of *B. montanum* root was validated for specificity, linearity, range, accuracy, precision, LOD and LOQ.

Specificity of each compound was performed by comparing retention time and UV spectrum of the peak in the chromatogram of crude extract with the respective peak in the chromatogram of standard solution. The linearity of each analyte was evaluated using the constructed calibration curve of serial concentrations of standard solutions. LODs and LOQs were investigated by using diluted standard solution. The signal to noise ratio (S/N) was calculated by dividing the peak height of the standard with the height of the background noise. The signal to noise (S/N) ratios calculated for LODs and LOQs were about 3 and 10, respectively.

Accuracy and precision of the method were evaluated by using three levels of spiked samples containing three concentrations of the standards. Crude extract solutions were spiked with the standard mixture solution of compound 1 (15, 25 and 40 μg/mL) and compound 2 (30, 50 and 80 μg/mL). Accuracy was calculated as percentage recovery. Intra-run and inter-run precision were calculated as RSD% of the concentration found from the subtracted spiked samples. The accuracy and precision of the method were determined by three replicates within-run for three consecutive runs.

### Statistical analysis

The results of the biological activities were reported as mean ± standard deviation (SD) from three replicated experiments. IC_50_ values were calculated by using a regression analysis. Mean differences among groups were analyzed by Student’s t-test. Statistical analysis was conducted by using GraphPad Prism software (CA, USA).

## Results

### Isolation and identification of pure compounds

**Fig. 1:**
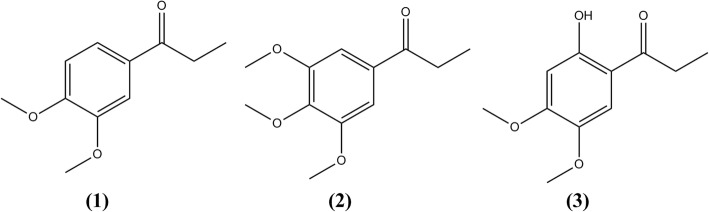
Chemical structures of propiophenons isolated from ethanolic extract of *B. montanum* root

Isolation of pure compounds from the ethanolic extract of *B. montanum* root produced three propiophenones (Fig. 1). The fractions obtained from the first CC were subjected to HPLC analysis to guide the isolation of major compounds (Additional file 1: Figure S1). FF03 and FF04 were combined based on the similarity of the HPLC profiles which contained two major compounds similar to the crude extract. The combined fraction was subjected to further silica gel CC. The fractions containing the major compounds (from the third silica gel CC) were subjected to the semi-preparative HPLC. Identification of the three isolated propiophenones was performed by spectrometric techniques (Additional file 1: Figure S2–S25 and Table S1–S3) with the following results.

Compound 1 was obtained as white solid (13.8 mg, 0.035% crude extract). The UV spectrum showed λ_max_ 229, 274, 303 nm. Positive ESI-HRMS at m/z of 217.0835 [M + Na]^+^ suggested the molecular formula to be C_11_H_14_O_3_Na. ^1^H NMR (500 MHz, CDCl_3_) *δ* 7.59 (H, dd, *J* = 8.4, 1.9 Hz), 7.52 (1H, d, *J* = 1.9 Hz), 6.88 (1H, d, *J* = 8.4 Hz), 3.93 (3H, s), 3.92 (3H, s), 2.95 (2H, q, *J* = 7.3 Hz), 1.21 (3H, t, *J* = 7.3 Hz). ^13^C NMR (125 MHz, CDCl_3_) *δ* 199.4, 153.3, 149.2, 130.4, 110.6, 110.3, 56.0, 31.3, 8.6. Based on ^1^H-^1^H COSY and HMBC correlations, compound 1 was suggested to be a known compound, 1-(3′,4′-dimethoxyphenyl) propan-1-one or propioveratrone. The structure was confirmed by comparing ^1^H-NMR and ^13^C-NMR spectral data to the data reported by Park and co-workers [15].

Compound 2 was obtained as white solid (30.1 mg, 0.075% *w*/w crude extract). The UV spectrum showed λ_max_ 217, 278 nm. Positive ESI-HRMS at m/z of 274.0941 [M + Na]^+^ suggested the molecular formula to be C_12_H_16_O_4_Na.^1^H NMR (500 MHz, CDCl_3_) *δ* 7.22 (2H, s), 3.93 (6H, s), 3.92 (3H, s), 2.97 (2H, *J* = 7.2 Hz), 1.22 (3H, *J* = 7.3 Hz). ^13^C NMR (125 MHz, CDCl_3_) *δ* 199.5, 153.2, 142.8, 132.3, 105.9, 60.9, 56.4, 31.5, 8.4. Based on ^1^H-^1^H COSY and HMBC correlations, compound 2 was suggested to be a known compound, 1-(3′,4′,5′-trimethoxyphenyl) propan-1-one. The structure was confirmed by comparing ^1^H-NMR and ^13^C-NMR spectral data to the data reported by Tanpure and co-workers [16].

Compound 3 was obtained as white solid (1.3 mg, 0.003% w/w crude extract). The UV spectrum showed λ_max_ 236, 275, 340 nm. Positive ESI-HRMS at m/z of 233.0784 [M + Na]^+^ suggested the molecular formula to be C_11_H_14_O_4_Na. ^1^H NMR (500 MHz, CDCl_3_) *δ* 12.75 (OH, s), 7.14 (1H, s), 6.45 (1H, s), 3.92 (3H, s), 3.84 (3H, s), 2.94 (2H, q, *J* = 7.3 Hz), 1.22 (3H, t, *J* = 7.3 Hz), 13C NMR (125 MHz, CDCl_3_) *δ* 204.8, 160.0, 156.5, 141.8, 111.2, 110.8, 100.7, 56.7, 56.2, 31.3, 8.6. Based on ^1^H-^1^H COSY and HMBC correlations, compound 3 was suggested to be a known compound 1-(2′-hydroxy- 4′, 5′-dimethoxyphenyl) propan-1-one. The structure was confirmed by comparing ^1^H-NMR and ^13^C-NMR spectral data to the data reported by Mendieta and co-workers [17].

### In vitro inhibitory effect on β-hexosaminidase release from RBL-2H3 cell lines

**Fig. 2:**
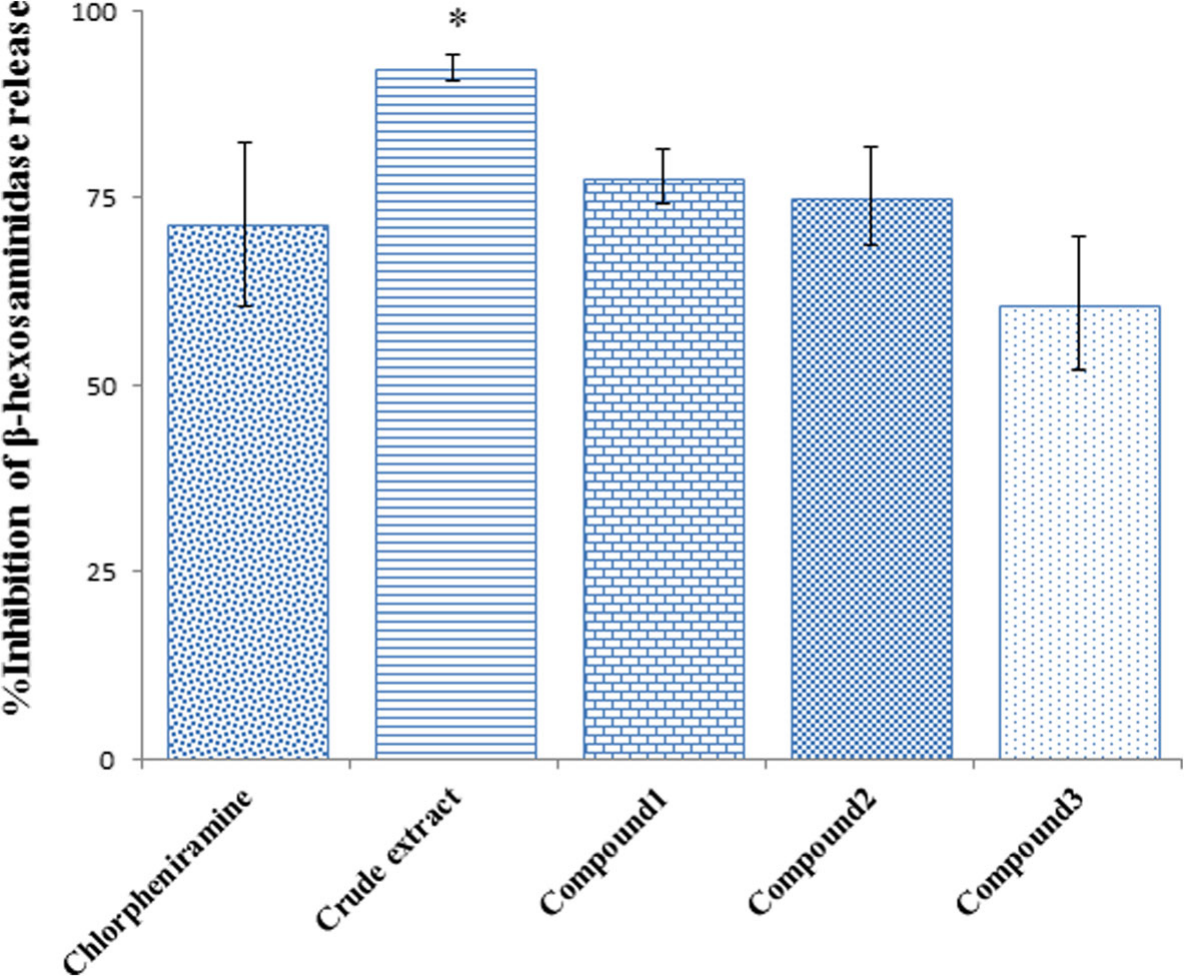
Effect of ethanolic extract of *B. montanum* root and the isolated propiophenones at concentration of 50 μg/mL on β-hexosaminidase release from RBL-2H3 cells. Results are present as mean ± SD from triplicate experiment. Mean differences between sample and positive control were analyzed by student’s t-test. *Significant difference comparing tested sample and positive control analyzed by Student’s t-test (*p*-value < 0.05)

β-hexosaminidase is a marker of IgE-mediated degranulation of mast cells relating to the symptoms of itching eczema [18]. Screening results of the anti-allergic activity against the releasing of β-hexosaminidase from RBL-2H3 cells of the crude extract and the isolated pure compounds at concentration 50 μg/mL are shown in Fig. 2. The crude extract exhibited significantly the highest activity while other compounds showed comparable %inhibition with the positive control, chlorpheniramine. These results indicated the possible anti-allergic activity of all investigated compounds and the crude extract. The IC_50_ investigation showed that all samples had concentration-dependent inhibitory effect (Table 1). The crude extract exhibited the highest anti-allergic activity with IC_50_ value of 4.97 ± 0.43 μg/mL which was three times more potent than the positive control (17.74 ± 1.61 μg/mL). The isolated compounds 1 and 2 showed anti-allergic activity comparable to the positive control with IC_50_ of 21.20 ± 2.01 μg/mL and 26.25 ± 6.06 μg/mL, respectively, while compound 3 showed moderate activity.

**Table 1:** Inhibitory effect of crude ethanolic extract and isolated compounds from *Baliospermum montanum* root on releasing of β-hexosaminidase from RBL-2H3 cell line

Sample	%Inhibition at various concentraction (μg/ml)					IC_50_ (μg/ml)
	0.1	1	10	50	100	
Crude extract	–	21.59 ± 0.59	81.77 ± 5.94	92.28 ± 1.84	94.13 ± 2.49	4.97 ± 0.43*
Compound 1	−7.88 ± 3.01	4.84 ± 3.46	29.40 ± 2.01	77.74 ± 3.60	–	21.20 ± 2.01
Compound 2	−5.37 ± 2.30	15.05 ± 1.71	30.37 ± 6.70	75.18 ± 6.47	–	26.25 ± 6.06
Compound 3	−14.21 ± 5.67	8.52 ± 2.93	17.38 ± 5.72	60.84 ± 8.98	–	44.42 ± 7.33*
Chlorpheniramine (Positive control)	–	14.89 ± 1.57	36.17 ± 2.84	71.43 ± 11.01	81.27 ± 9.51	17.74 ± 1.61

Data performed as mean ± SD from triplicate experiments. Mean difference of IC_50_ data of tested sample and positive control were compared by Student’s t-test. * Significant difference at *p-*value < 0.05

### In vitro inhibitory activity of NO production from LPS-induced RAW 264.7

**Fig. 3:**
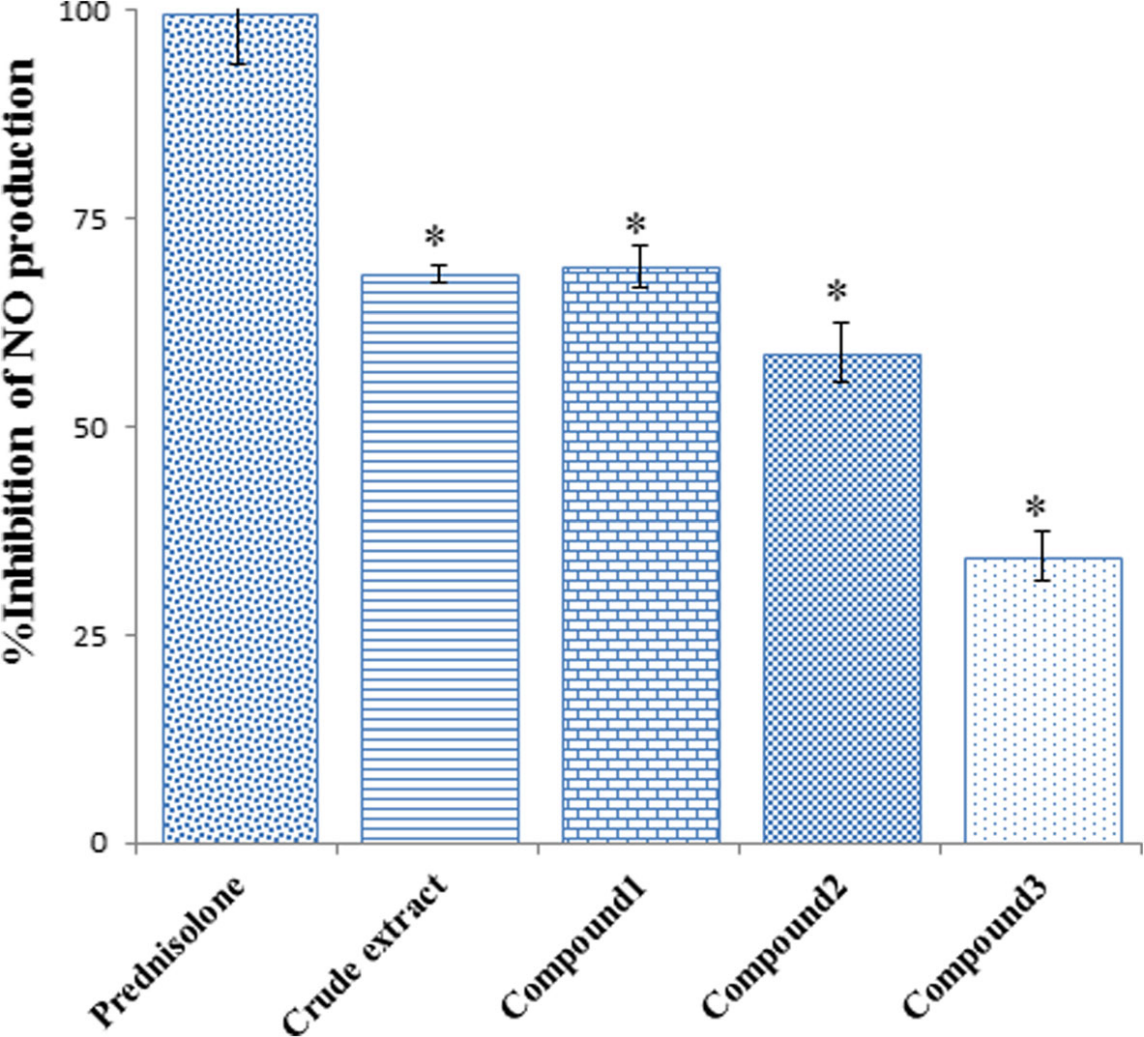
Effect of ethanolic extract of *B. montanum* root and the isolated propiophenones at concentration of 50 μg/mL on NO production from RAW 264.7 cells. Results are presented as mean±SD from triplicate experiment. Mean differences between sample and positive control were analyzed by student’s t-test. *Significant difference comparing tested sample and positive control analyzed by Student’s t-test (*p*-value < 0.05)

NO is a pro-inflammatory mediator released from macrophage during inflammation. Excessive concentration of NO production from inducible NO synthase (iNOS) in macrophage is linked to inflammatory reactions. NO has an important role along with several mediators in inflammatory diseases including rheumatoid arthritis and osteoarthritis [19, 20]. The screening results of the inhibitory activity of NO production of the crude extract and its isolated compounds at the concentration of 50 μg/mL are presented in Fig. 3. The crude extract and all compounds exhibited %inhibition less than prednisolone (positive control). The crude extract, compounds 1 and 2 showed comparable %inhibition while compound 3 showed the least inhibitory effect. From the determination of IC_50_, the crude extract and isolated compounds inhibited the production of NO in a concentration-dependent manner (Table 2). The crude extract exhibited the highest inhibitory activity on NO production with IC_50_ of 6.06 ± 0.43 μg/mL while the isolated compounds showed moderate activity with IC_50_ more than 30 μg/mL. Among the three isolated propiophenones, compounds 2 exhibited the highest inhibitory activity with IC_50_ of 33.61 ± 5.25 μg/mL. All compounds and crude extract showed less inhibitory activity than prednisolone which exhibited potent inhibitory activity with IC_50_ value of 0.11 ± 0.02 μg/mL.

**Table 2:** Inhibitory effect of isolated compounds on *LPS*-induced nitric oxide release from RAW 264.7 cell lines

Sample	%Inhibition at various concentration (μg/mL) (%Viability)					IC_50_(μg/mL)
	0.01	0.1	1	10	50	
Crude extract	–	16.65 ± 6.18 (92.51 ± 2.14)	32.42 ± 5.42 (89.44 ± 3.74)	54.64 ± 2.23 (83.49 ± 2.66)	68.38 ± 1.04 (78.82 ± 2.83)	6.06 ± 0.43*
Compound 1	–	2.66 ± 0.23 (84.66 ± 7.91)	3.91 ± 3.03 (89.71 ± 12.08)	14.41 ± 1.71 (84.71 ± 9.65)	69.15 ± 2.49 (80.46 ± 3.10)	40.40 ± 1.32*
Compound 2	–	7.09 ± 6.86 (94.52 ± 10.55)	11.12 ± 9.89 (88.81 ± 8.07)	8.69 ± 6.93 (80.85 ± 7.39)	58.83 ± 3.59 (90.39 ± 9.92)	33.61 ± 5.25*
Compound 3	–	−0.82 ± 3.26 (102.40 ± 2.17)	2.64 ± 0.31 (96.95 ± 0.98)	14.59 ± 3.43 (97.27 ± 4.43)	34.44 ± 3.07 (91.62 ± 3.87)	> 50*
Prednisolone (Positive control)	19.82 ± 3.43 (100.40 ± 12.94)	47.72 ± 6.24 (84.28 ± 4.56)	69.94 ± 3.76 (89.46 ± 2.28)	77.94 ± 8.12 (80.78 ± 2.71)	–	0.11 ± 0.02

Data performed as mean ± SD from triplicate experiments. Mean difference of IC_50_ data of tested sample and positive control were compared by Student’s t-test. *Significant difference at *p-*value < 0.05

### In vitro cytotoxic activity

Cytotoxic activity of the *B.montanum* root extract and isolated compounds were investigated against selected two cancerous liver cells, HepG2 and KKU-M156, and a normal cell, HaCaT. All samples exhibited cytotoxic activity in direct proportion to their concentrations (data not shown). Their IC_50_ are presented in Table 3. The crude extract exhibited potent cytotoxic activity against HepG2 and KKU-M156 with IC_50_ less than 1 μg/mL. Interestingly, crude extract exhibited less cytotoxicity to HaCaT with the IC_50_ more than 100 μg/mL. However, the isolated compounds exhibited moderate cytotoxic activity to the cancerous cells with IC_50_ more than 45 μg/mL. Though the crude extract and isolated compounds exhibited cytotoxic activity less than positive control (vincristine), the cytotoxic effect of the extract and compounds against normal cells were lower compared to their effect against cancerous cells, showing their selective cytotoxic property.

**Table 3:** Cytotoxic activity of the crude ethanolic extract and the isolated compounds against cancerous liver cells (HepG2 and KKU-M156) and normal cells (HaCaT)

Sample	IC_50_ (μg/mL) (Selective index)		
	HepG2	KKU-M156	HaCaT
Crude extract	0.06 ± 0.02* (> 100)	0.16 ± 0.02* (> 100)	> 100*
Compound1	87.07 ± 1.36* (> 1.15)	58.32 ± 5.95* (> 1.71)	> 100*
Compound2	47.61 ± 1.20* (> 2.10)	95.65 ± 0.48* (> 1.04)	> 100*
Compound3	90.33 ± 5.65* (> 1.11)	83.38 ± 5.20* (> 1.20)	> 100*
Vincristine	0.013 ± 0.0006 (< 0.008)	0.0030 ± 0.0015 (< 0.033)	< 0.0001

Data performed as mean ± SD from triplicate experiments. Mean difference of IC_50_ data of tested sample and positive control were compared by Student’s t-test. *Significant difference at *p-*value < 0.05

### HPLC method development and method validation

**Fig. 4:**
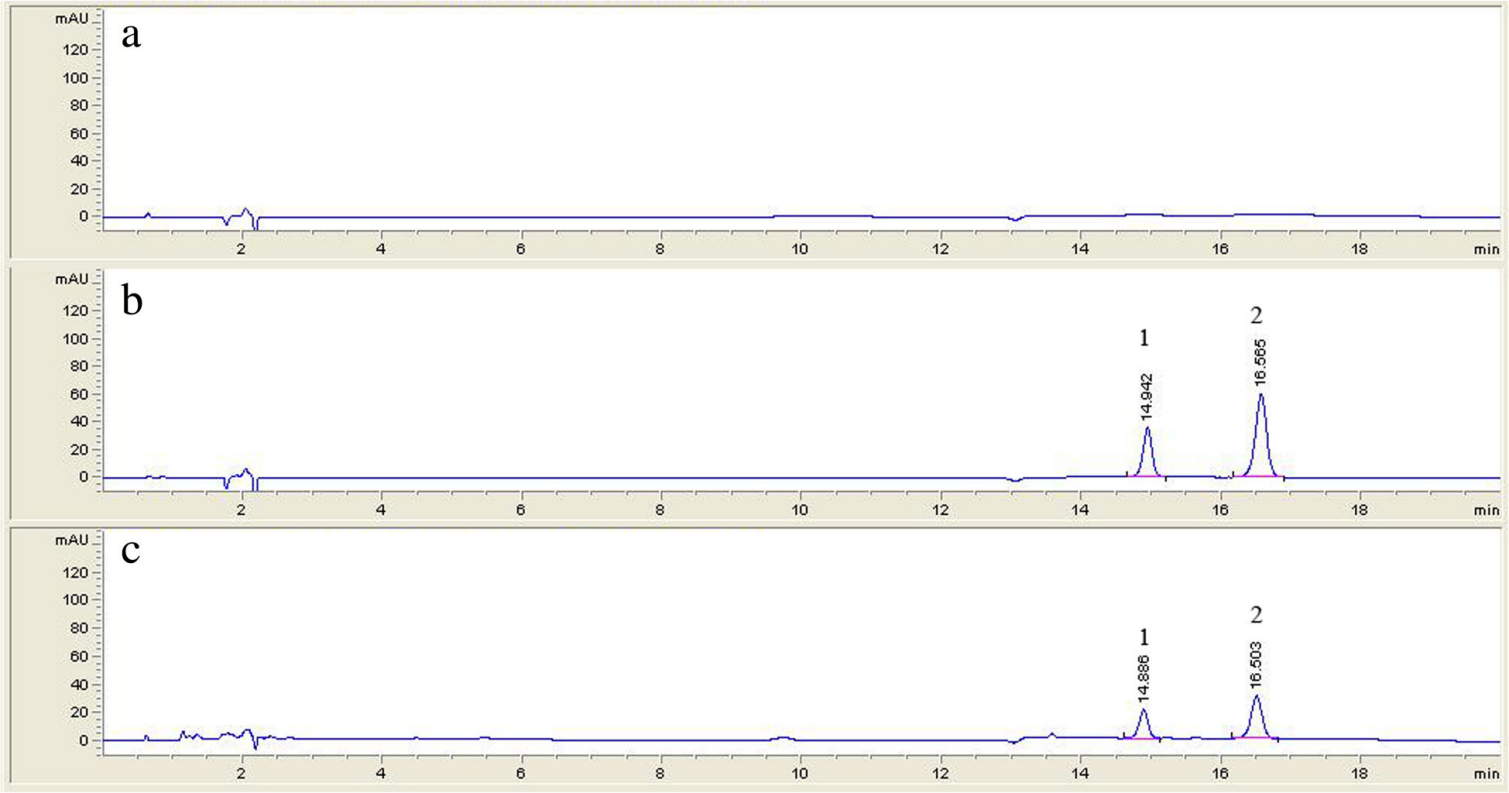
HPLC chromatogram of blank (methanol)) (**a**), standard solution (10 μg/mL) (**b**) and crude extract solution in methanol (4 mg/mL) (**c**)

**Fig. 5:**
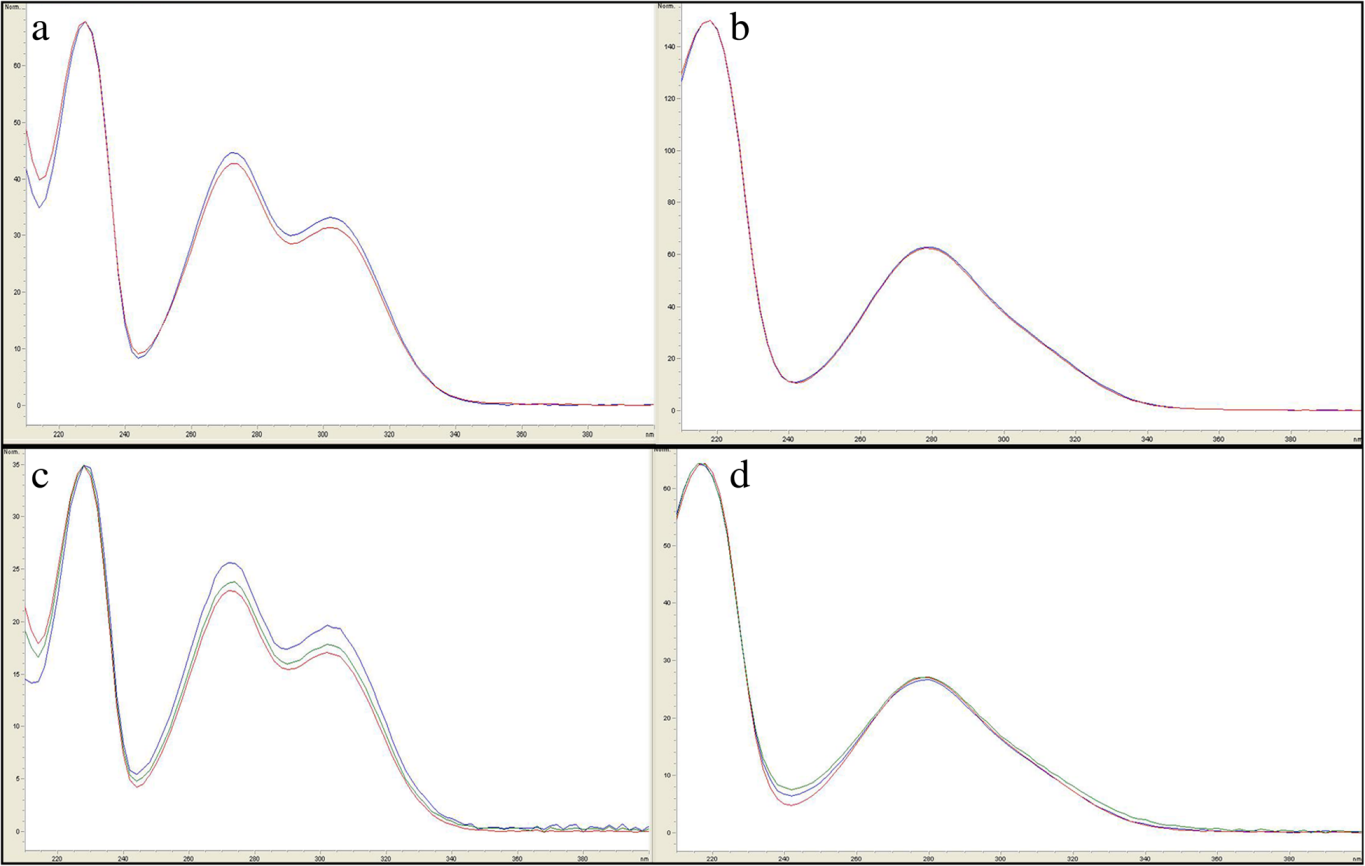
Overlay UV spectra of compound 1 (**a**) and compound 2 (**b**) in crude extract solution (4 mg/mL) compared with respective standards in standard solution (10 μg/mL); overlay UV spectrum of each compound at peak start, peak apex and peak end: compound 1 (**c**) and compound 2 (**d**)

In the three isolated propiophenones, compound 1 and compound 2 were the major peaks found in the HPLC chromatogram of the ethanolic extract of *B. montanum* root, therefore, we used these two compounds as markers for quantitative HPLC analysis. Compound 3 showed very small amount and its potency on the biological activities were lower than those of the others. The selectivity of the method is shown in Fig. 4. Compounds 1 and 2 showed the retention times of about 14.9 min and 16.6 min, respectively. No peaks were found in the chromatogram of the blank (methanol) at the same retention times as the standards. The UV spectra of the peaks of compounds 1 and 2 in the chromatogram of the crude extract corresponded to the respective standards in the chromatogram of standard solutions (Fig. 5a, b). Peak purity of each compound in sample chromatogram was evaluated by comparing the UV spectra at peak start, peak apex and peak end which were similar (Fig. 5c, d). The linearity, range, LOD, and LOQ of compounds 1 and 2 are shown in Table 4.

**Table 4:** Linearity, range, LOD and LOQ of compound 1 and compound 2

Compounds	Validation parameters			
	Linearity	Range (μg/mL)	LOD (μg/mL)	LOQ (μg/mL)
Compound 1	Y = 29.32x + 10.02; r^2^ = 0.9998	5–50	0.05	0.2
Compound 2	Y = 30.71x + 31.60; r^2^ = 1	10–100	0.05	0.2

LOD and LOQ of both compounds 1 and 2 were 0.05 μg/mL and 0.2 μg/mL, respectively. The method showed good linearity demonstrated by the coefficient of determination (r^2^) being more than 0.999 within the ranges of 5 μg/mL - 50 μg/mL, for compound 1 and 10 μg/mL - 100 μg/mL for compound 2. The accuracy of both compounds are presented as %recovery as shown in Table 5. The recovery of both compounds were within 97.7–103.0%. The precision shown as RSD% was evaluated both intra-run and between-run. The RSD% of each compound was less than 2.4% for both within-run and between-run showing good precision of the method. The developed HPLC method showed good linearity, accuracy, and precision and, hence, can be used to determine the major components of the ethanolic extract of *B. montanum* root.

**Table 5:** Accuracy and precision of quantitative method for determination of compound 1 and compound 2 in ethanolic extract of root of *B.montanum*

Compounds	Spiked concentration (μg/mL)	Concentration found (μg/mL; Mean ± SD)	RSD	%Accuracy
Intra-run (n = 3)				
Compound 1	15	14.94 ± 0.32	2.1	98.3–102.0%
	25	24.97 ± 0.38	1.5	
	40	40.65 ± 0.39	1.0	
Compound 2	30	29.63 ± 0.11	0.4	98.3–102.3%
	50	50.04 ± 1.18	2.4	
	80	81.24 ± 1.63	2.0	
Inter-run (*n* = 9)				
Compound 1	15	14.91 ± 0.20	1.3	97.7–102.0%
	25	24.87 ± 0.29	1.2	
	40	40.43 ± 0.44	1.1	
Compound 2	30	29.83 ± 0.40	1.3	98.0–103.0%
	50	50.19 ± 0.87	1.7	
	80	81.38 ± 1.03	1.3	

## Discussion

Evidential uses of traditional herbal medicines are established by ancient doctors in several societies around the world. However, scientific information on these herbal medicines is required to either support or dispute their traditional uses. As the interest in alternative medicines grows, the knowledge accumulated over the years by the traditional doctors can be used to guide modern scientists along the right path to discovery. In the present study, we set out to study the root of *B. montanum*, which has been used in TTM as an ingredient in remedies for the treatments of itching eczema, muscle and joint inflammation and cancer (especially liver cancer). Crude ethanolic extract of the root was fractionated, major components of each fraction were determined for which an analytical method was developed and its efficacy verified, followed by the analysis of biological activities of the crude extract and its components against the targeted diseases.

The crude ethanolic extract exhibited potent inhibitory effects in all biological activities investigated. It showed three times higher anti-allergic activity than the positive control, chlorpheniramine. Venkatesh and co-workers, using in vivo anaphylaxis model, have also shown anti-allergic effects of the leaves of *B. montanum* [21]. For anti-inflammatory activity, the crude extract inhibited the inducible NO production from RAW 264.7 cells with IC_50_ comparable to that reported by Kakatum and co-workers [5]. In cytotoxic study, the crude extract exhibited selective cytotoxic activity against the two cancerous liver cells, HepG2 and KKU-M156 but not normal cell, HaCaT. There are some evidences of cytotoxic effects of *B. montanum* against HT-29 human colon cancer [22] and PC3 prostate cancer [23]. Cherian and co-workers reported the evidence of selective cytotoxic effects of *B. montanum* on PC3 prostate cells but not on normal mouse embryonic fibroblast, NIH3T3 [23]. United States National Cancer Institute (NCI) has developed criteria for evaluating potential cytotoxic plants which states that plant extracts should have IC_50_ less than 30 μg/mL when incubated with cancer cells for 48–72 h to be considered viable [24]. Therefore, the *B. montanum* root crude extract which showed toxicity against the cancerous liver cells in this study appears to fit that definition. However, further study with normal liver cells is needed to prove its selective cytotoxicity.

The crude ethanolic extract of the *B. montanum* root showed potential effects on immune and inflammatory responses. In TTM, *B. montanum* has been prescribed to patients with different symptoms (itching eczema, muscle and joint inflammation, cancer). However, the pathologies of these symptoms are related to a variety of cytokines, chemokines, mediators and cells [25]. Therefore, their inter-relationship should also be a subject for further studies.

Isolation of major constituents from the *B. montanum* root revealed three propiophenones. The isolated propiophenones exhibited the highest anti-allergic activity followed by anti-inflammatory activity and cytotoxicity. Compounds 1 and 2 exhibited comparable potency in all activities while compound 3 exhibited the lowest effect. All isolated compounds can inhibit the proliferation of cancerous cells in a concentration-dependent fashion, however, their potency was only moderate to low. Venkatesh and co-workers reported that acridanone alkaloids isolated from the leaves of *B. montanum* inhibited the degranulation of mast cells in systemic anaphylaxis model in vivo [7]. Ogura and co-workers reported cytotoxicity of phorbol type diterpenes isolated from petroleum ether extract and chloroform-soluble fractions of *B. montanum* root [6]. GCMS analysis of leaves of *B. montanum* revealed five antimicrobial terpenoids from acidified ethyl acetate extract [26]. It appears that our study is the first to isolate propiophenones from the *B. montanum* root and to report their biological activities. The isolated propiophenones exhibited only moderate effects in all tested activities, and the fact that the crude extract showed greater potency than its isolated compounds may be due to the multicomponent synergistic effects which have also been shown in several studies [27–29]. However, to prove the synergistic effects, not only major components but also minor ones should be isolated and investigated for their biological activities.

Propiophenones are a group of organic chemicals widely used as precursors of organic synthesis experiments or occur as byproducts. In nature, propiophenones can be found in some plants such as *Acorus gramineus* (Araceae) [15], and *Trigonostemon howii* (Euphorbiaceae) [30]. Their skeletal structure composes of a benzene ring connecting to three carbon propene tails similar to phenylpropanoids. In the present study, compound 3 which possesses a hydroxyl group at 2′-carbon on the aromatic ring, exhibited the lowest biological activities among the three compounds. Thus, this hydroxyl group may not be a necessary component for these activities. In structure-activity relationship (SAR) studies of the compounds with similar skeletal structure, phenylpropanoids, the substitution of acetoxyl group at 4′ and 1 positions of phenylpropanoids (adjusted nomenclature similar to our study) are required for the strong inhibitory effects on β-hexosaminidase released from RBL-2H3 [31] and cytotoxic effects against MDA-MB-231 breast cancer cells [32]. For the inhibitory activity of NO production, the methoxyl group at 4′*-*position and the hydroxyl group at 3- position with one glucopyranosyl moiety in the molecule are required [33]. This may be concluded that the isolated propiophenones cannot be considered responsive compounds for the investigated activities.

The only report on isolation of components from the root of *B. montanum* by Ogura and co-workers showed that the isolated phorbol diterpenes from its hexane and chloroform-soluble extracts exhibited potent cytotoxic effects against leukemia P388 cells [6]. However, phorbol diterpenes may be minor components in the ethanolic extract. Therefore, to detail the responsive markers of the crude ethanolic extract of *B. montanum* root, other minor constituents should be isolated and investigated further to evaluate their roles in the cytotoxic activities.

A HPLC method was developed for the determination of major components of *B. montanum* root extract. Compounds 1 and 2 were found to be major components of the extract from the HPLC chromatogram. The developed method showed good linearity, accuracy, and precision. The method may be useful for further study on the formulation development of the remedies and for quality control in the production of the crude extract. Propiophenones, compounds 1 and 2, analyzed with the HPLC method may be used as markers to indicate the presence of *B. montanum* root in multi-ingredients traditional remedies. A more sensitive analytical method such as LCMSMS may also be used in conjunction to detect minor components that affect biological activities of the extract.

## Conclusion

The results of this study reveal potential benefits of *B. montanum* root as a natural source of anti-allergy, anti-inflammation, and anti-cancer ingredient. Three propiophenones were isolated from its crude ethanolic extract for the first time. The crude ethanolic extract of *B. montanum* root exhibited potent biological activities while its isolated major components exhibited only moderate activities. A HPLC method was developed and validated for the analysis of the major components of the extract with the results showing good linearity, precision, and accuracy. The results from this study support the use of the root of *B. montanum* in traditional medicines and provide novel information on its biological activities and chemical components. However, the major compounds isolated may not be responsible for all its biological activities; isolation and study of minor components are necessary to evaluate their roles in its overall effectiveness. The biological activities that indicate the relationship between immunity, inflammation, and cancer should also be investigated. A more sensitive analytical method should be developed for detailing the key responsive components in the extract.

## Additional file

Additional file 1: **Figure S1.** HPLC profiles of obtained fractions from first CC comparing with crude extract. **Figure S2-S25.** Structural formulae, spectrometric data and HPLC chromatograms of the three isolated propiophenones. **Table S1-S3.** 1H NMR and 13C NMR chemical shift data of the three isolated propiophenones. (DOCX 6268 kb)

## Abbreviations

Anti-DNP IgE: Anti-dinitrophenyl immunoglobulin E
BSA: Bovin serum albumin
CaCl_2_: Calcium chloride
CC: Column chromatography
CO_2_: Carbon dioxide
COSY: Correlated Spectroscopy
DMEM: Dulbecco’s Modified Eagle’s Medium
DMSO: Dimethyl sulfoxide
DNP-BSA: Anti-dinitrophenylated bovine albumin
ESI-HRMS: Electrospray ionization- high resolution mass spectrometry
FBS: Fetal bovine serum
HAM F-12: Nutrient Mixture F-12 Ham medium
HCl: Hydrochloric acid
HMBC: Heteronuclear multiple bond correlation
HPLC: High performance liquid chromatography
IC_50_: Half inhibitory concentration
IT-TOF: Ion trap-time of fight spectrometer
KCl: Potassium chloride
LOD: Limit od detection
LOQ: Limit of quantitation
LPS: Lipopolysaccharide
MEM: Minimum essential medium
MgCl_2_: Magnesium chloride
MS: Masspectrometry
MTT: 3-(4, 5-dimethyl-2-thiazolyl)-2, 5-diphenyl-2H-tetrazolium bromide
NaCl: Sodium chloride
NaOH: Sodium hydroxide
NMR: Nuclear magnetic resonance
NO: Nitric oxide
P/S: Penicillin-streptomycin
PBS: Phosphate buffer saline
PIPES: Piperazine-N,N′-bis (2-ethanesulfonic acid
PNAG: 4-Nitrophenyl N-acetyl-β-D-glucosaminide
RPMI: Roswell Park Memorial Institute medium 1640
S/N: Signal to noise
SRB: Sulforhodamine B
TLC: Thin layer chromatography
TTM: Thai traditional medicines
