# Supplementary material for: Potential in vitro anti-allergic, anti-inflammatory and cytotoxic activities of ethanolic extract of Baliospermum montanum root, its major components and a validated HPLC method
Source: BMC Complement Altern Med. 2019 Feb 12;19:45. doi: 10.1186/s12906-019-2449-0 (PMC6373163; doi:10.1186/s12906-019-2449-0)
Supplement: Supplementary file 1 — Figure S1. HPLC profiles of obtained fractions from first CC comparing with crude extract. Figure S2-S25. Structural formulae, spectrometric data and HPLC chromatograms of the three isolated propiophenones. Table S1-S3. 1H NMR and 13C NMR chemical shift data of the three isolated propiophenones. (DOCX 6268 kb) [file 12906_2019_2449_MOESM1_ESM.docx]

**Additional File 1**

**Potential *in vitro* anti-allergic, anti-inflammatory and cytotoxic activities of ethanolic extract of *Baliospermum montanum* root, its major components and a validated HPLC method**

Weerachai Pipatrattanaseree^1,2^, Arunporn Itharat^1,2*^, Nichamon Mukkasombut^1,2^, Ubonwan Saesiw^1,2^

^1^Department of Applied Thai Traditional Medicine, Faculty of Medicine, Thammasat University, Klongluang, Pathumthani, 12120, Thailand.

^2^Center of Excellence on Applied Thai Traditional Medicine Researches, Faculty of Medicine, Thammasat University, Klongluang, Pathumthani, 12120, Thailand.

**Corresponding author:**

Associated Professor Arunporn Itharat, Ph,D.

Department of Applied Thai Traditional Medicine, Faculty of Medicine, Thammasat University, Klongluang, Pathumthani, 12120, Thailand.

Email: [iarunporn@yahoo.com](mailto:iarunporn@yahoo.com)

**
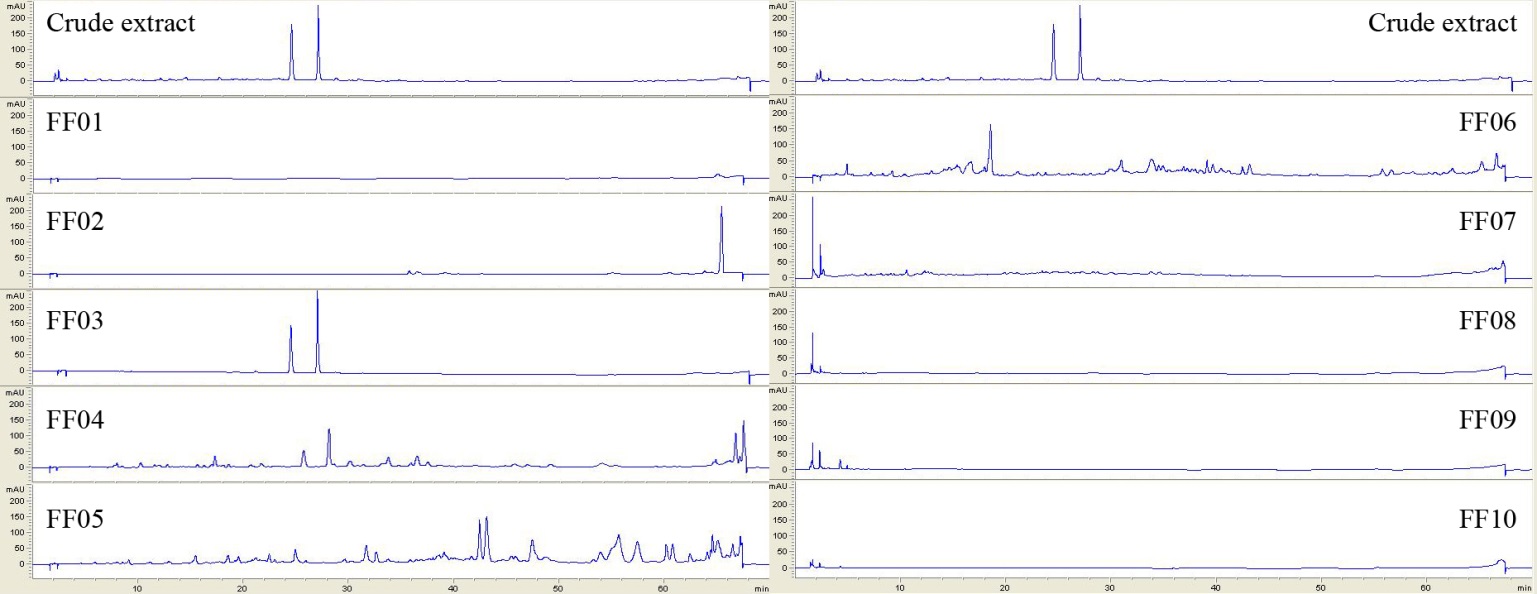
**

**Figure S1.** HPLC profiles of obtained fractions from first CC comparing with crude extract.

HPLC profiles of fractions from first column (FF01 – FF10) were analyzed along a reverse phase analytical column (Zorbax® XDB-C18, 4.6 mm. x 250 mm., 5 µm). Gradient mobile phase composed of water (A) and acetonitrile (B) were programed as follows: 0-35 min, 10%B - 50%B; 35 – 55 min, 50%B - 60%B; 60 min - 65 min, 70%B - 95%B; 65.1 min - 70 min, 10%B. The flow rate was set at 1.0 ml/min. Samples were injected into the HPLC system and detected with diode array detector using wavelength 280 nm.

**Compound 1**

**Figure S2** Structural formula of 1-(3’,2',4^'^-dimethoxyphenyl) propan-1-one **(Compound 1)**


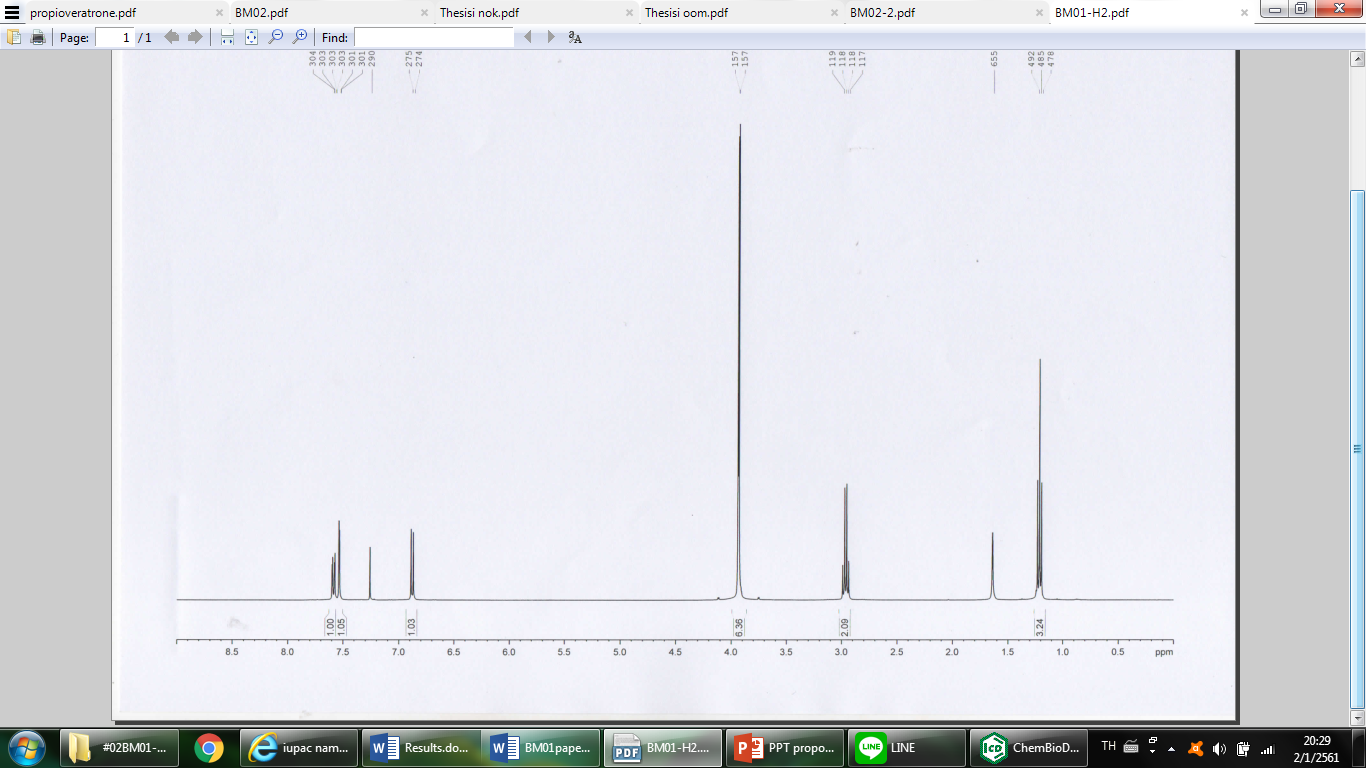


**Figure S3** ^1^H NMR spectrum of **Compound 1** (500 MHz, CDCl_3_)


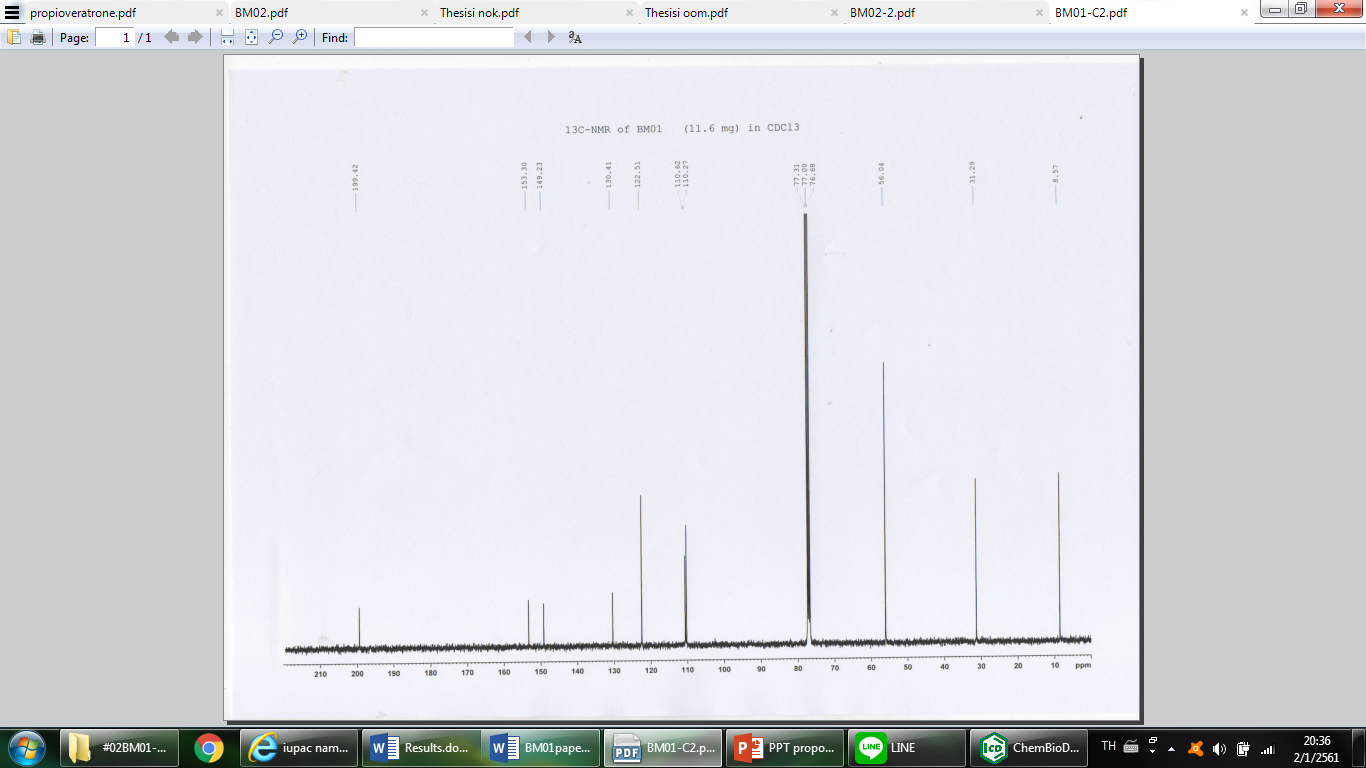


**Figure S4**  ^13^C NMR spectrum of **Compound 1** (125 MHz, CDCl_3_)

**Table S1** ^1^H NMR and ^13^C NMR of 1-(3^/^,4^/^--dimethoxyphenyl) propan-1-one (**Compound 1).**

| Position | *δ_C_*  Experiment | *δ_C_*  [15] | *δ_H_* (*J* in Hz)  Experiment | *δ_H_* (*J* in Hz)  [15] |
| --- | --- | --- | --- | --- |
| 1 | 199.4, C | 199.7, C | - | - |
| 2 | 31.3, CH_2_ | 31.5, CH_2_ | 2.95, q (7.3; 2H) | 2.96, q (7.2; 2H) |
| 3 | 8.6, CH_3_ | 8.6, CH_3_ | 1.21, t (7.3; 3H) | 1.22, t (7.0; 3H) |
| 1^/^ | 130.4, C | 130.4, C | - | - |
| 2^/^ | 110.3, C | 110.2, C | 7.52, d (1.9; 1H) | 7.54, s, 1H |
| 3^/^ | 149.2, C | 149.3, C | - | - |
| 4^/^ | 153.3, C | 153.3, C | - | - |
| 5^/^ | 110.6, C | 110.5, C | 6.88, d (8.4; 1H) | 6.88, d (8.4; 1H) |
| 6^/^ | 122.5, C | 122.7, C | 7.59, dd (8.4, 1.9; 1H) | 7.58, d (8.0; 1H) |
| 3^/^-OCH_3_ | 56.0, 2CH_3_ | 56.2, CH_3_ | 3.92, s, 3H | 3.94, s, 6H |
| 4^/^-OCH_3_ |  | 56.3, CH_3_ | 3.93, s, 3H |  |


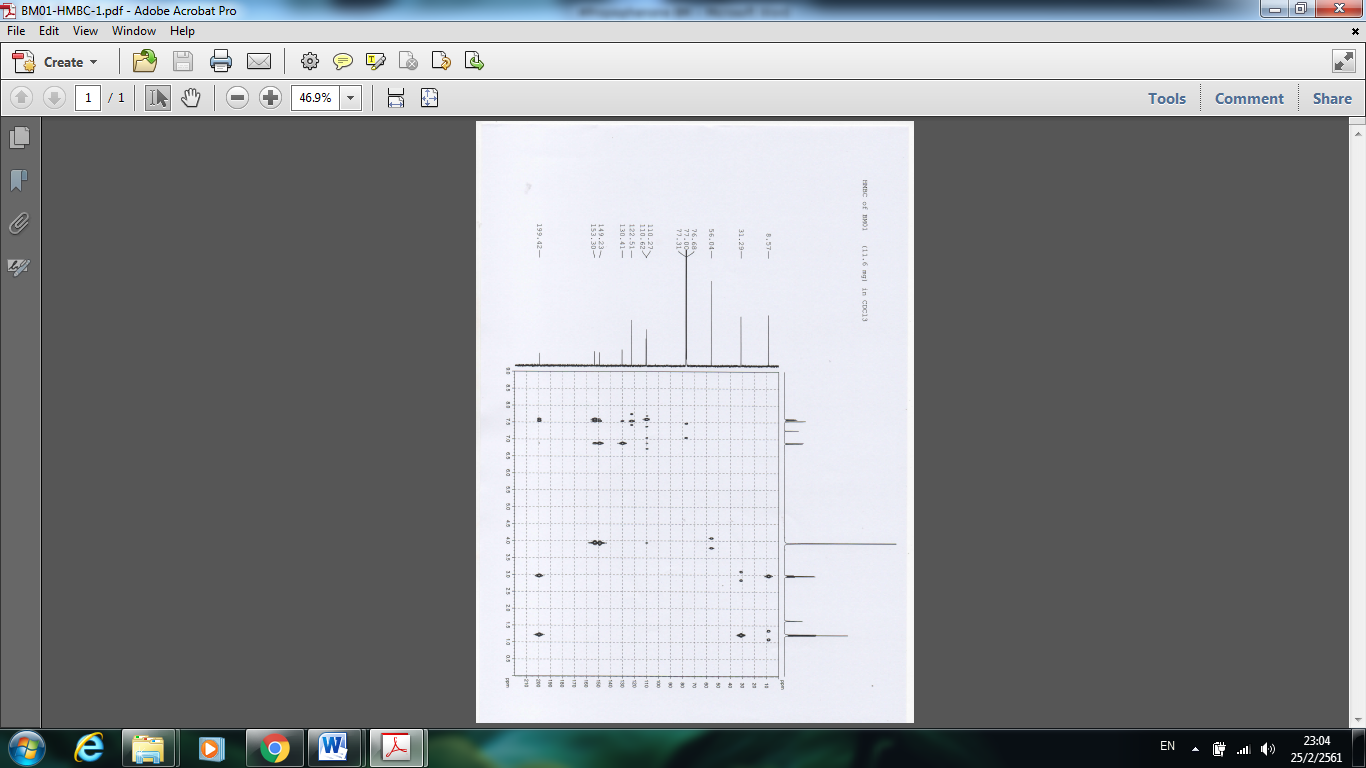


**Figure S5** HMBC correlations of **Compound 1**


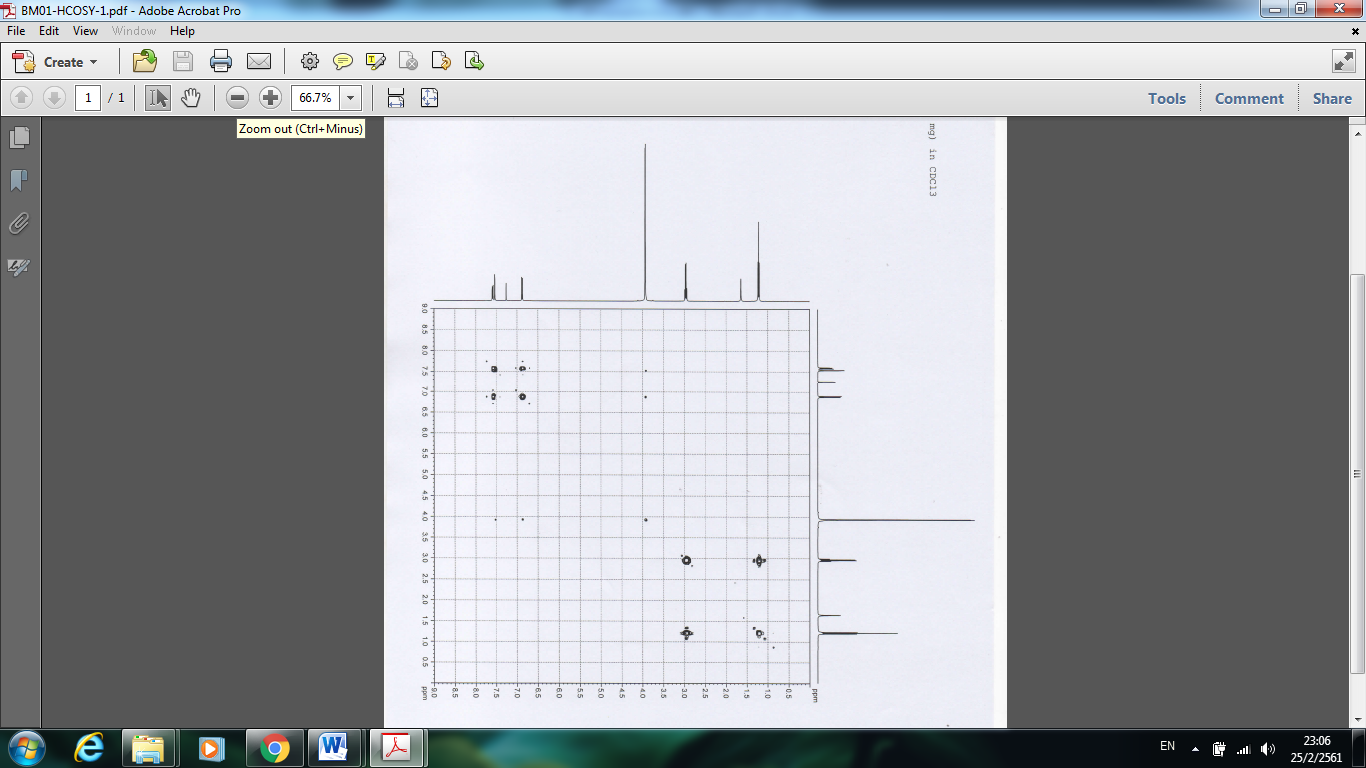


**Figure S6** ^1^H-^1^H COSY correlation of **Compound 1**


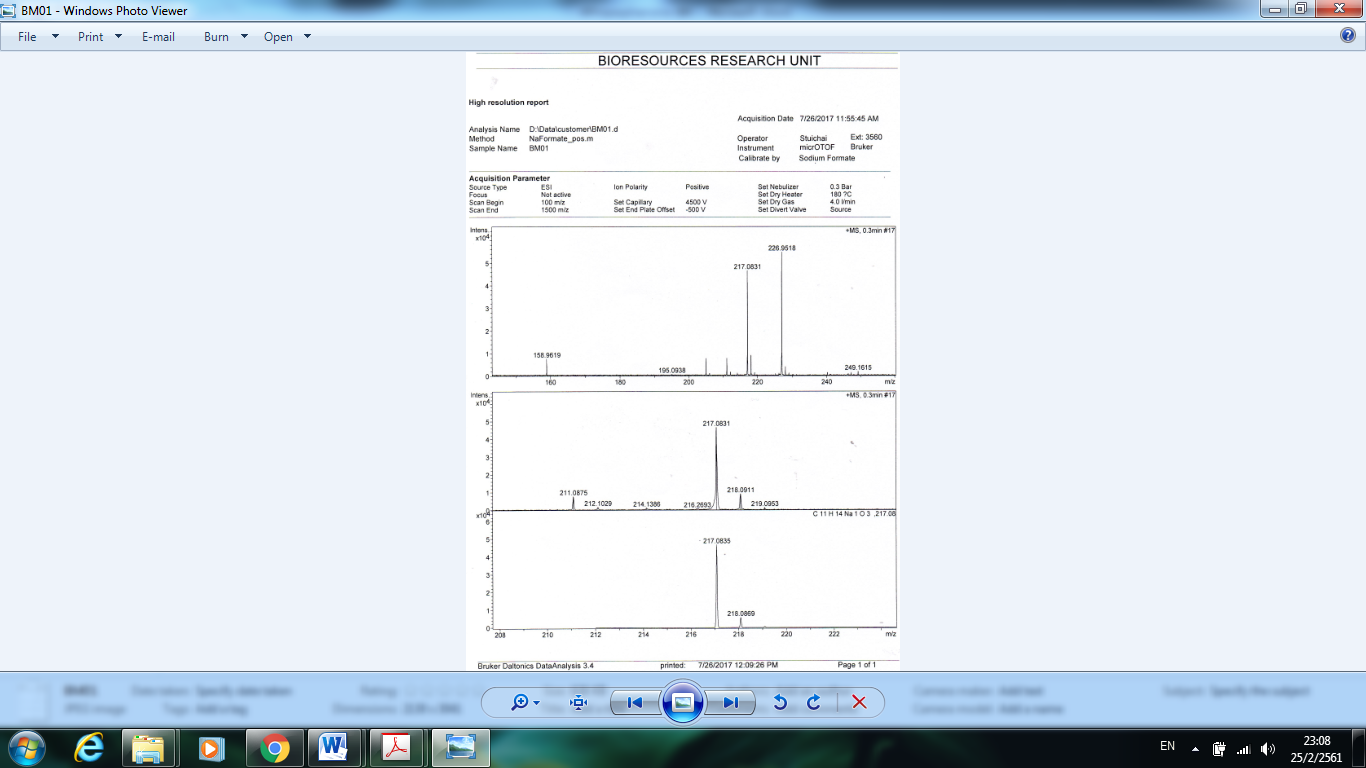


**Figure S7** HR-ESI-MS of **Compound 1**


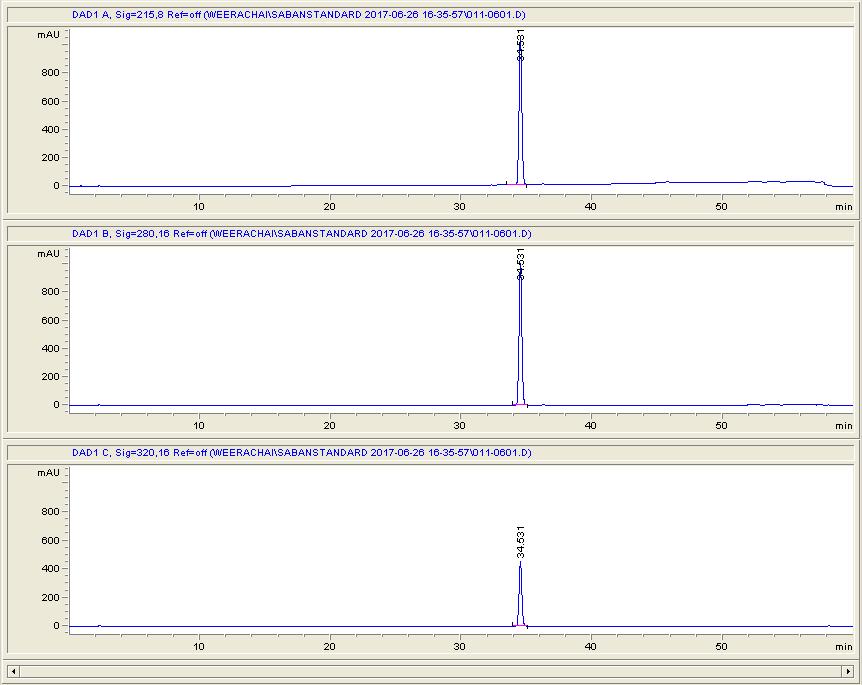


**Figure S8** HPLC chromatogram of **Compound 1** (1 mg/mL) at various wavelengths.


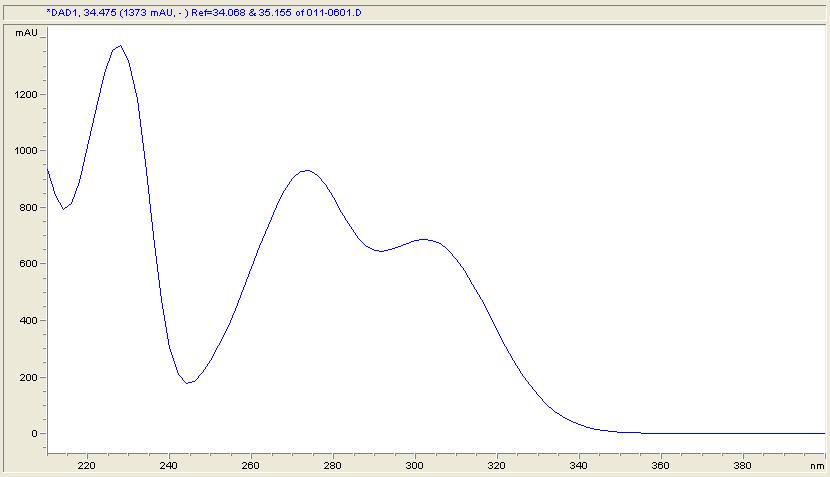


**Figure S9** UV spectrum of **Compound 1** (1 mg/mL)

**Compound 2**

**Figure S10** Structural formula of 1-(3^/^,4^/^,5^/^-dimethoxyphenyl) propan-1-one

(**Compound 2**)


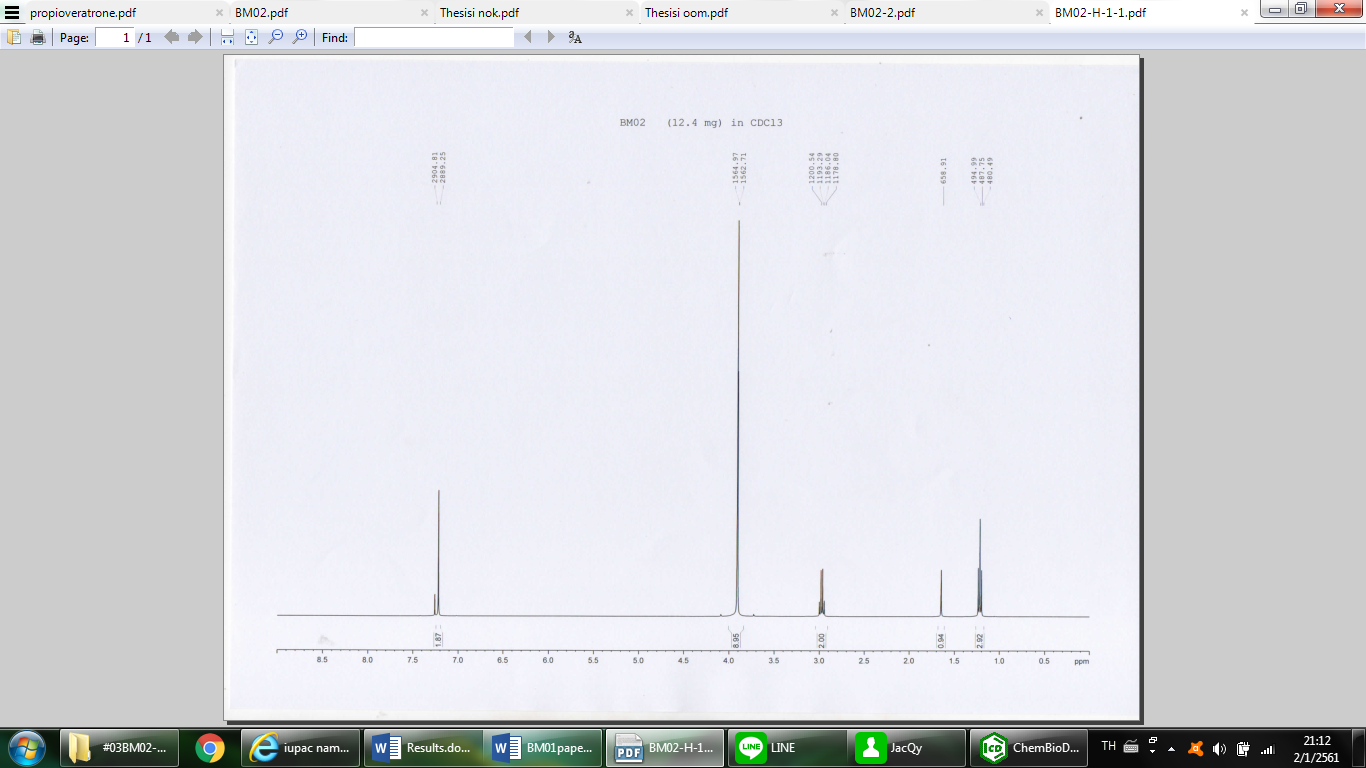


**Figure S11** ^1^H NMR spectrum of **compound 2** (500 MHz, CDCl_3_)


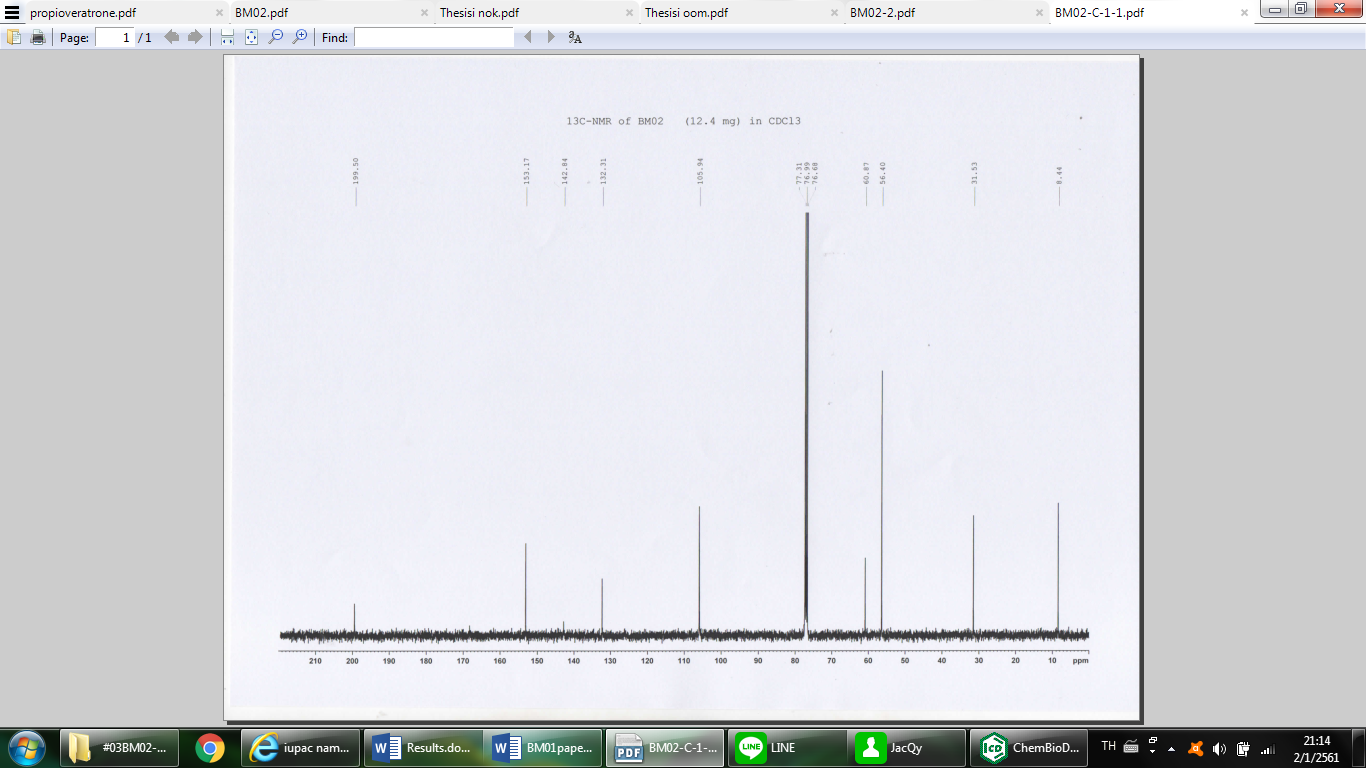


**Figure S12** ^13^C NMR spectrum of **compound 2** (125 MHz, CDCl_3_)

**Table S2** ^1^H NMR and ^13^C NMR of 1-(3^/^,4^/^,5^/^-dimethoxyphenyl) propan-1-one **(Compound 2)**

| Position | *δ_C_*  Experiment | *δ_C_*  [16] | *δ_H_* (*J* in Hz)  Experiment | *δ_H_* (*J* in Hz)  [16] |
| --- | --- | --- | --- | --- |
| 1 | 199.5, C | 199.6, C | - | - |
| 2 | 31.5, CH2 | 31.6, CH2 | 2.97, q (7.2; 2H) | 2.98, q (7.5; 2H) |
| 3 | 8.4, CH3 | 8.4, CH3 | 1.22, t (7.3; 3H) | 1.23, t (7.5; 3H) |
| 1^/^ | 132.3, C | 132.2, C | - | - |
| 2^/^, 6^/^ | 105.9, 2C | 105.5, 2C | 7.22, s, 2H | 7.22, s, 2H |
| 3^/^, 5^/^ | 153.2, 2C | 153.0, 2C | - | - |
| 4^/^ | 142.8, C | 142.4, C | - | - |
| 3^/^-OCH_3_,  5^/^-OCH_3_ | 56.4, 2CH3 | 56.3, 2CH3 | 3.93, s, 6H | 3.93, s, 6H |
| 4^/^-OCH_3_ | 60.9, CH3 | 60.9, CH3 | 3.92, s, 3H | 3.92, s, 3H |


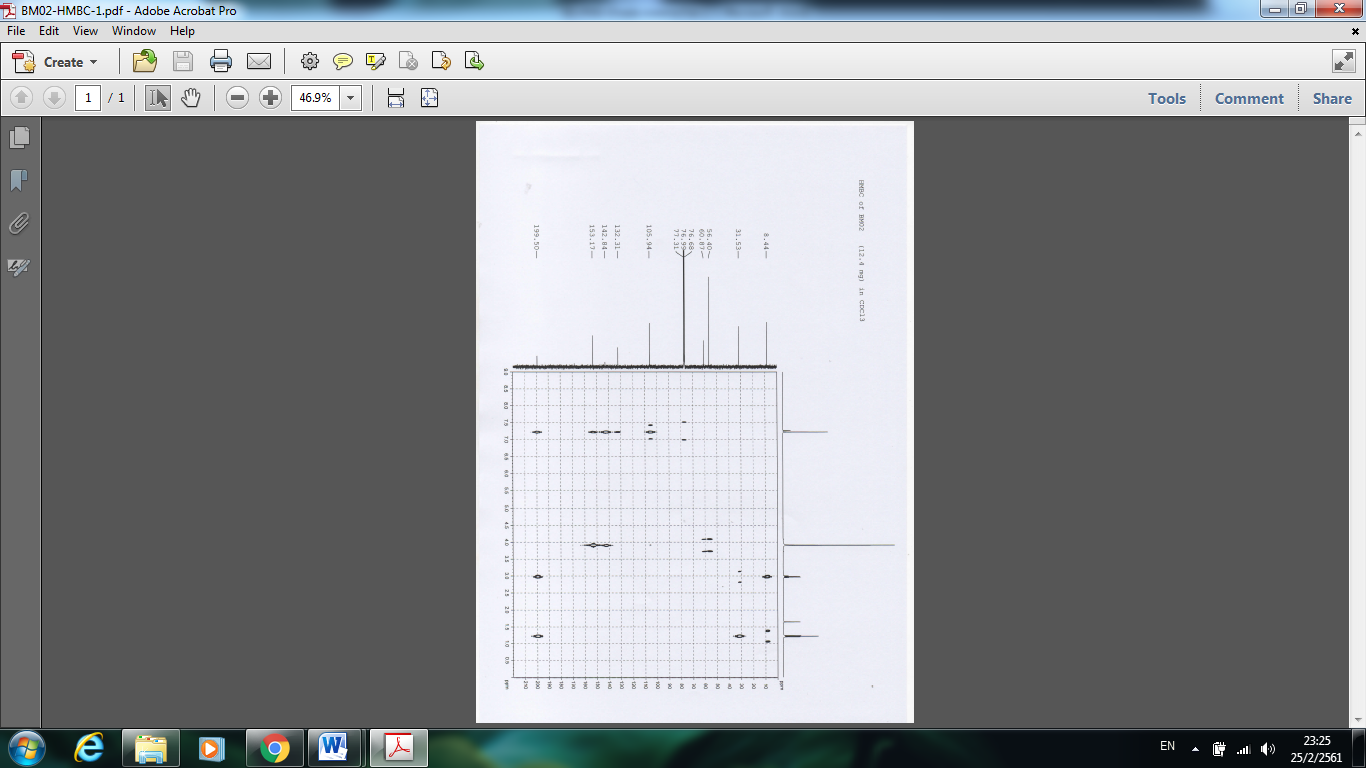


**Figure S13** HMBC correlations of **Compound 2**


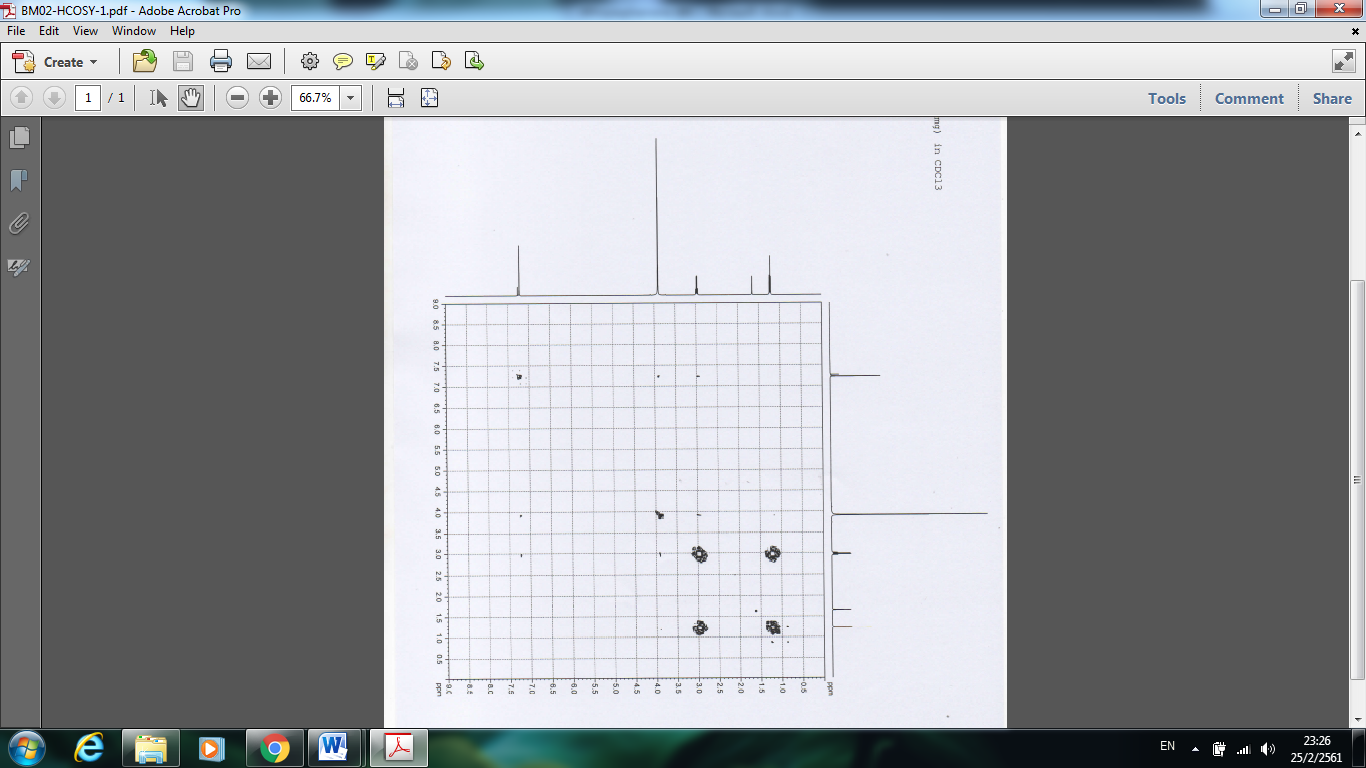


**Figure S14** ^1^H-^1^H COSY correlation of **Compound 2**


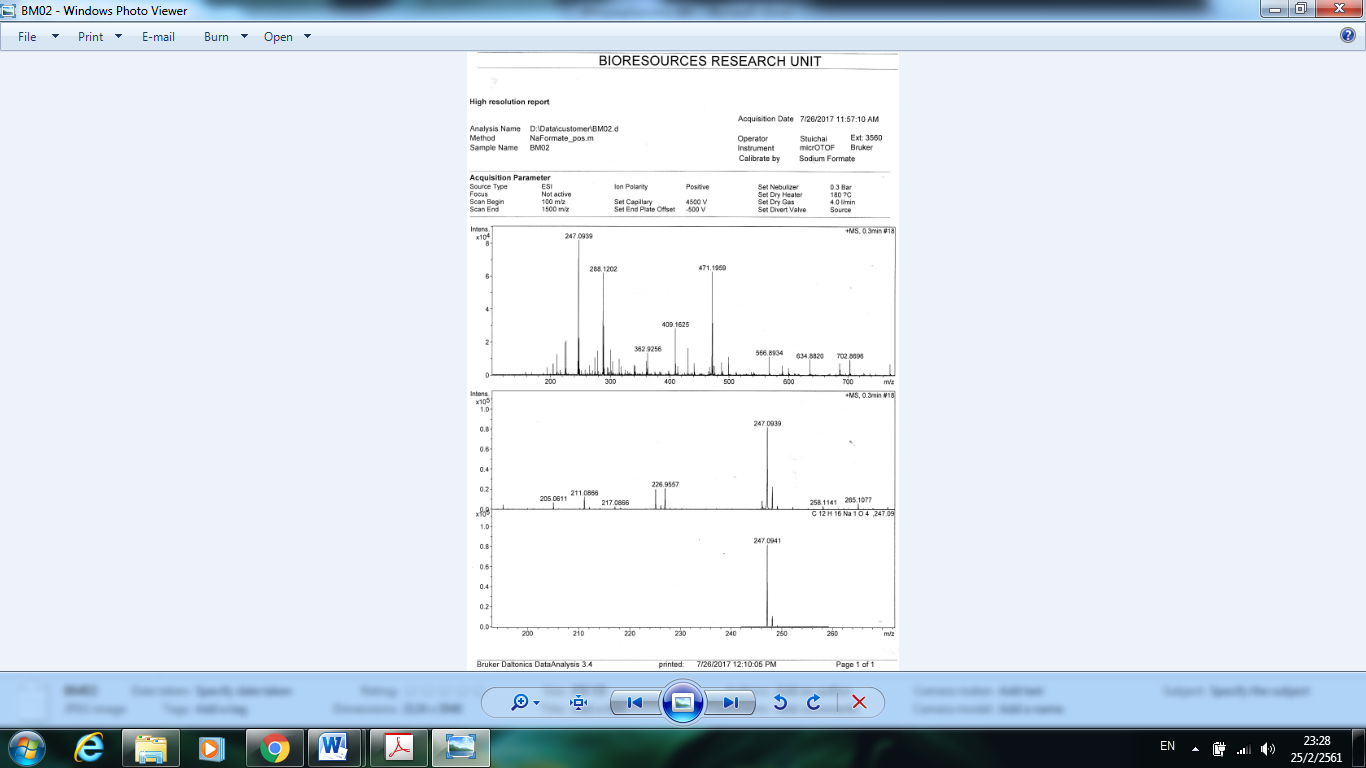


**Figure S15** HR-ESI-MS of **Compound 2**


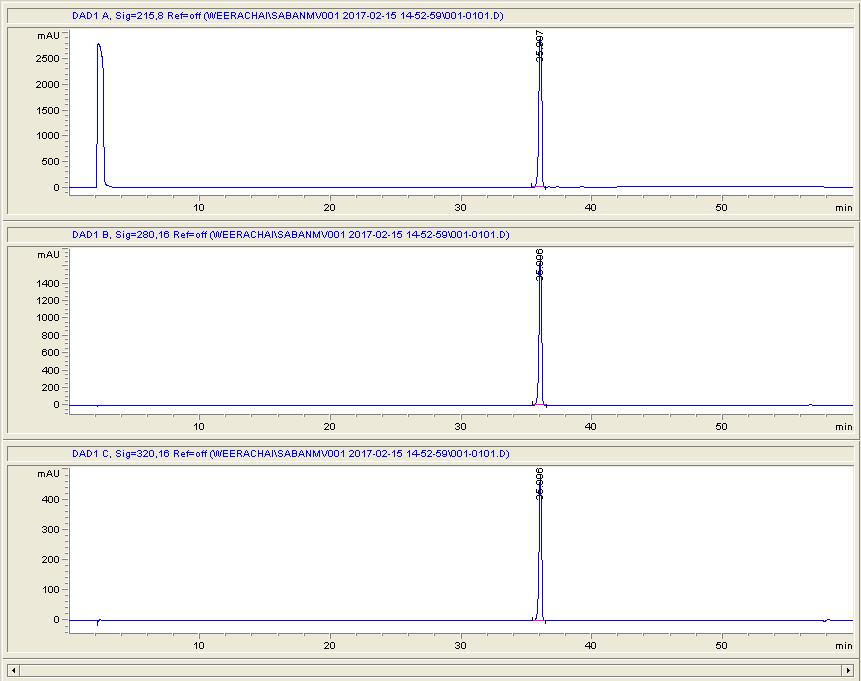


**Figure S16** HPLC chromatogram of **Compound 2** (1 mg/mL) at various wavelengths.


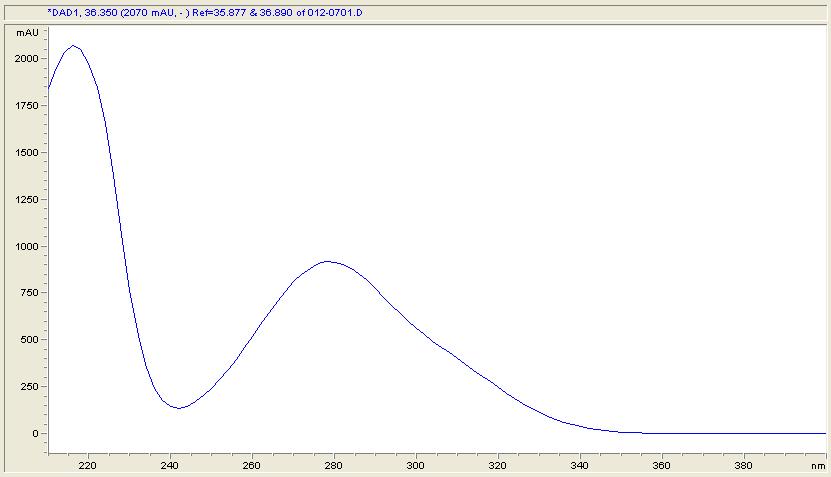


**Figure S17** UV spectrum of **Compound 2** (1 mg/mL)

**Compound 3**

**Figure S18.** Structural formula of **1-(2^/^-hydroxy- 4^/^, 5^/^-dimethoxyphenyl)**

**propan-1-one** (**Compound 3**)


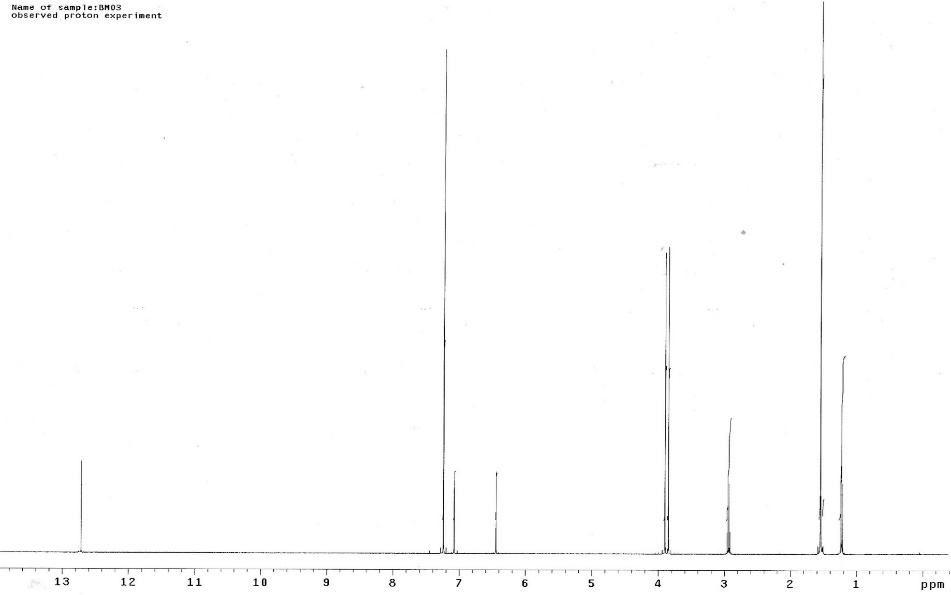


**Figure S19** ^1^H NMR spectrum of **Compound 3** (500 MHz, CDCl_3_)


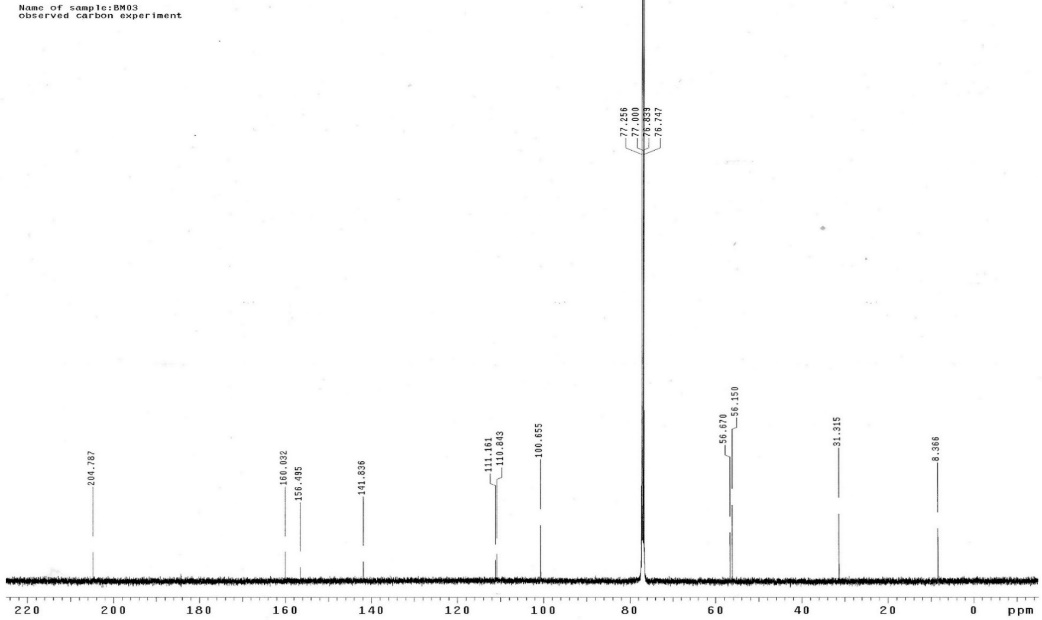


**Figure S20** ^13^C NMR spectrum of **BM03** (125 MHz, CDCl_3_)

**Table S3** ^1^H NMR and ^13^C NMR of 1-(2^/^-hydroxy- 4^/^, 5^/^-dimethoxyphenyl) propan-1-one

**(Compound 3)**

| Position | *δ_C_*  Experiment | *δ_C_*  [17] | *δ_H_* (*J* in Hz)  Experiment | *δ_H_* (*J* in Hz)  [17] |
| --- | --- | --- | --- | --- |
| 1 | 204.8, C | 204.7, C | - | - |
| 2 | 31.3, CH_2_ | 31.3, CH_2_ | 2.94, q (7.3; 2H) | 2.95, q (7.5; 2H) |
| 3 | 8.4, CH_3_ | 8.3, CH_3_ | 1.22, t (7.3; 3H) | 1.24, t (7.5; 3H) |
| 1^/^ | 110.8, C | 110.9, C | - | - |
| 2^/^ | 160.0, C | 160.0, C | - | - |
| 3^/^ | 100.7, CH | 100.6, CH | 6.45, s | 6.46, s |
| 4^/^ | 156.5, C | 156.5, C | - | - |
| 5^/^ | 141.8, C | 141.8, C | - | - |
| 6^/^ | 111.2, CH | 111.1, CH | 7.14, s | 7.10, s |
| 2^/^-OH | - | - | 12.75, s, OH | 12.76, s, OH |
| 4^/^-OCH_3_ | 56.2, CH_3_ | 56.1, CH_3_ | 3.84, s, 3H | 3.86, s, 3H |
| 5^/^-OCH_3_ | 56.7, CH3 | 56.7, CH3 | 3.92, s, 3H | 3.91, s, 3H |

**
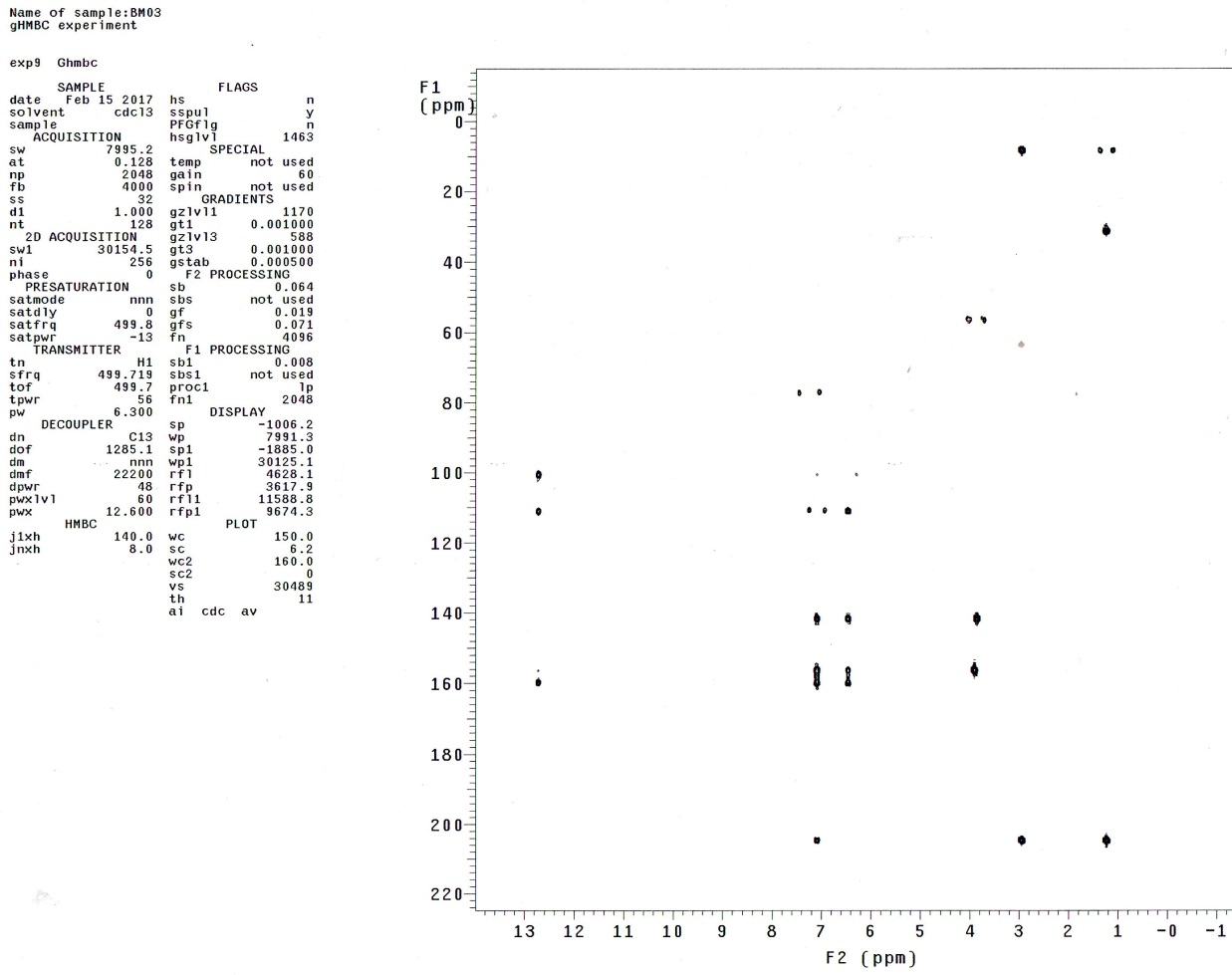
**

**Figure S21** HMBC correlations of **Compound 3**

**
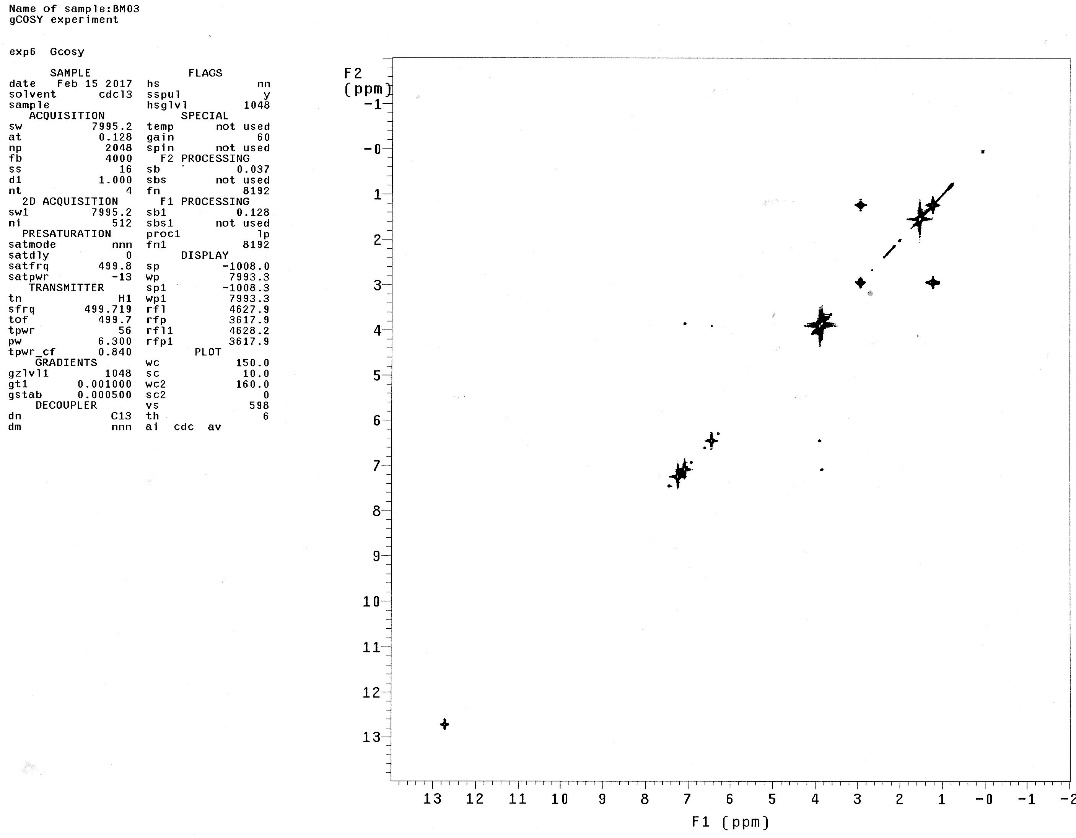
**

**Figure S22** ^1^H-^1^H COSY correlation of **Compound 3**

**
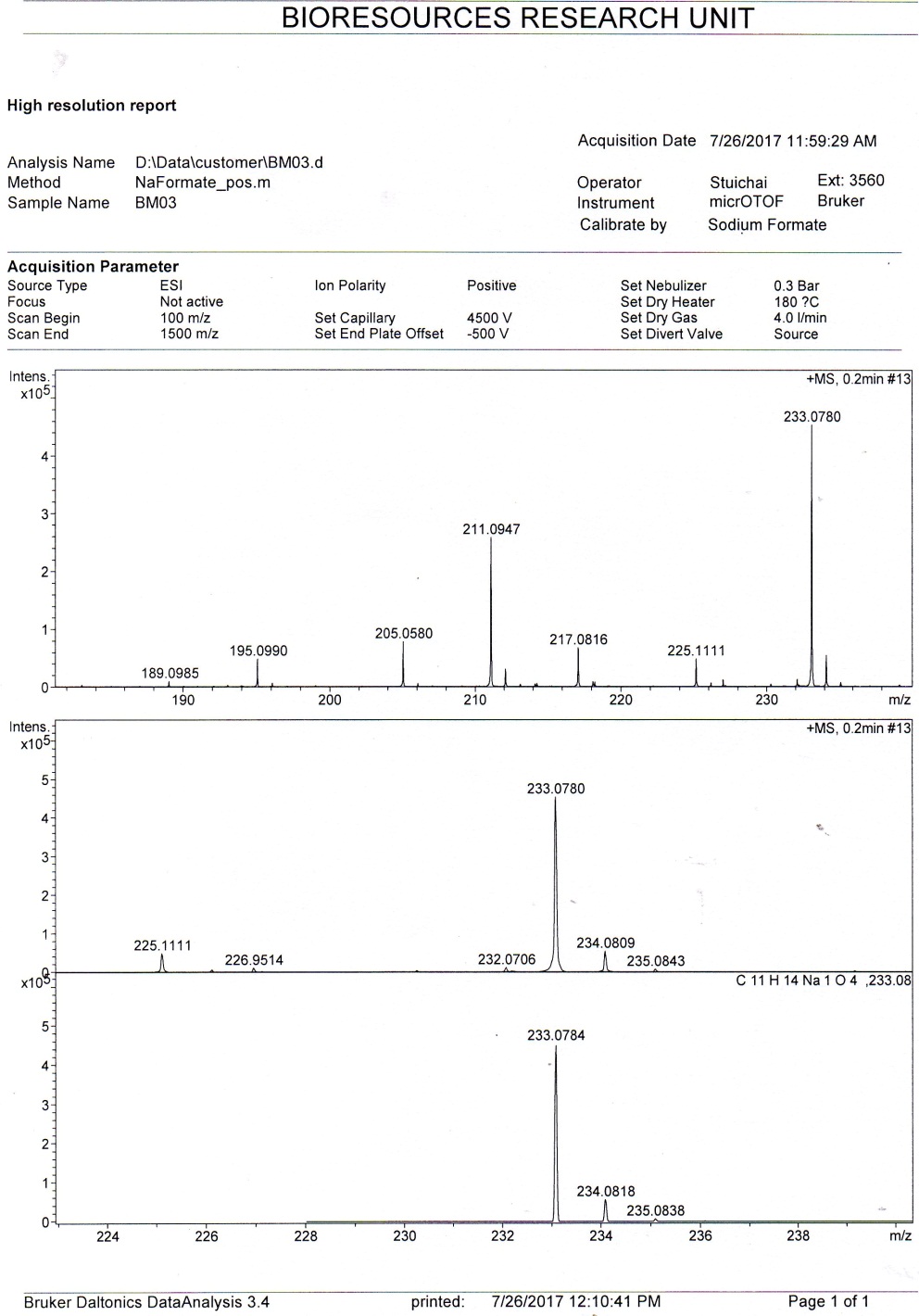
**

**Figure S23** HR-ESI-MS of **Compound 3**

**
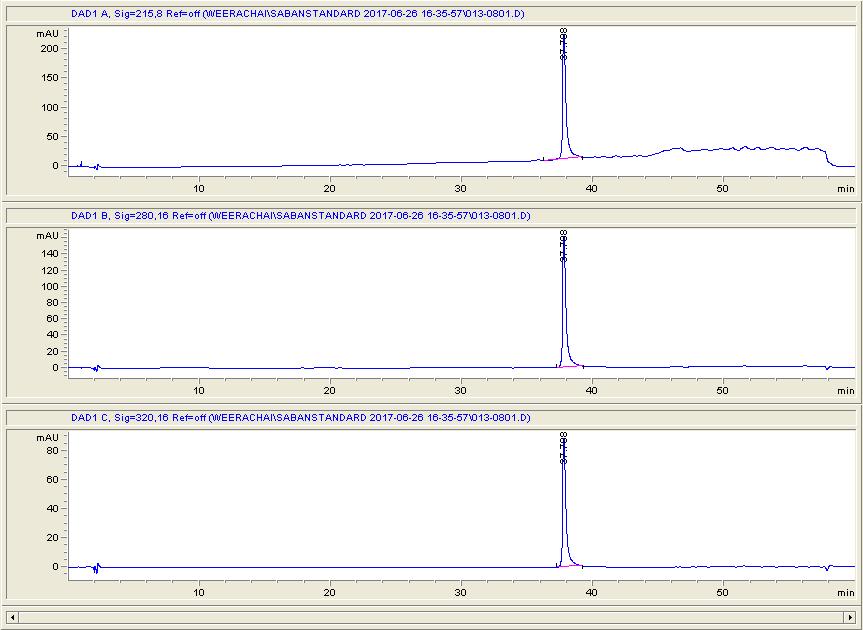
**

**Figure S24** HPLC chromatogram of **Compound 3** (0.2 mg/mL) at various wavelengths.

**
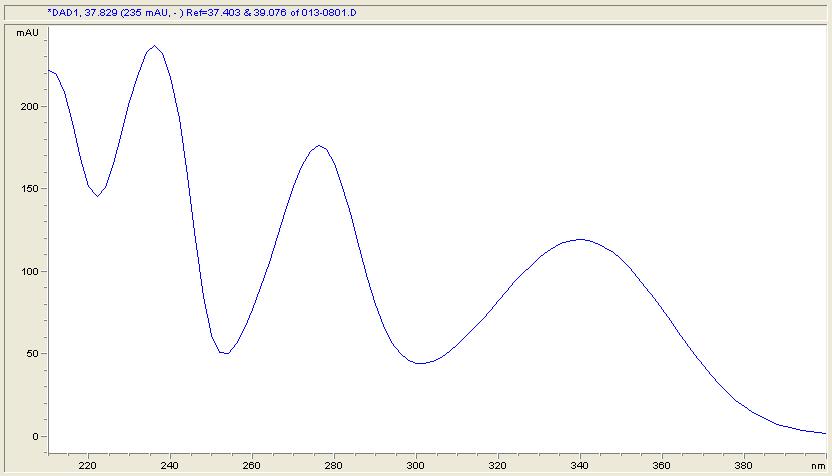
**

**Figure S25** UV spectrum of **Compound 3** (0.2 mg/mL)
